# MicroRNAs Are Intensively Regulated during Induction of Somatic Embryogenesis in Arabidopsis

**DOI:** 10.3389/fpls.2017.00018

**Published:** 2017-01-23

**Authors:** Katarzyna Szyrajew, Dawid Bielewicz, Jakub Dolata, Anna M. Wójcik, Katarzyna Nowak, Aleksandra Szczygieł-Sommer, Zofia Szweykowska-Kulinska, Artur Jarmolowski, Małgorzata D. Gaj

**Affiliations:** ^1^Department of Genetics, Faculty of Biology and Environmental Protection, University of SilesiaKatowice, Poland; ^2^Department of Gene Expression, Faculty of Biology, Institute of Molecular Biology and Biotechnology, Adam Mickiewicz UniversityPoznan, Poland

**Keywords:** Arabidopsis, gene expression, mature miRNA, *MIRNA* genes, pri-miRNA, somatic embryogenesis

## Abstract

Several genes encoding transcription factors (TFs) were indicated to have a key role in the induction of somatic embryogenesis (SE), which is triggered in the somatic cells of plants. In order to further explore the genetic regulatory network that is involved in the embryogenic transition induced in plant somatic cells, micro-RNA (miRNAs) molecules, the products of *MIRNA* (*MIR*) genes and the common regulators of TF transcripts, were analyzed in an embryogenic culture of *Arabidopsis thaliana*. In total, the expression of 190 genes of the 114 *MIRNA* families was monitored during SE induction and the levels of the primary (pri-miRNAs) transcripts vs. the mature miRNAs were investigated. The results revealed that the majority (98%) of the *MIR* genes were active and that most of them (64%) were differentially expressed during SE. A distinct attribute of the *MIR* expression in SE was the strong repression of *MIR* transcripts at the early stage of SE followed by their significant up-regulation in the advanced stage of SE. Comparison of the mature miRNAs vs. pri-miRNAs suggested that the extensive post-transcriptional regulation of miRNA is associated with SE induction. Candidate miRNA molecules of the assumed function in the embryogenic response were identified among the mature miRNAs that had a differential expression in SE, including miR156, miR157, miR159, miR160, miR164, miR166, miR169, miR319, miR390, miR393, miR396, and miR398. Consistent with the central role of phytohormones and stress factors in SE induction, the functions of the candidate miRNAs were annotated to phytohormone and stress responses. To confirm the functions of the candidate miRNAs in SE, the expression patterns of the mature miRNAs and their presumed targets were compared and regulatory relation during SE was indicated for most of the analyzed miRNA-target pairs. The results of the study contribute to the refinement of the miRNA-controlled regulatory pathways that operate during embryogenic induction in plants and provide a valuable platform for the identification of the genes that are targeted by the candidate miRNAs in SE induction.

## Introduction

Somatic embryogenesis (SE) reflects the unique developmental potential of plant somatic cells, which results in the transition of the differentiated somatic cells that are cultured *in vitro* into the embryogenic ones that form the somatic embryos. Thus, studies on SE provide basic knowledge about the molecular and genetic mechanisms that govern the developmental plasticity in plants. It is believed that genes that have a regulatory function activated by plant growth regulators and stress that is imposed *in vitro* play a key role in the mechanism of embryogenic transition (Jiménez, 2005; Karami and Saidi, 2010). In line with this assumption, numerous genes encoding transcription factors (TFs) were indicated as being involved in the regulatory pathway that operates in SE induction, including *LEAFY COTYLEDON2* (*LEC2*) (Gaj et al., 2005; Ledwoń and Gaj, 2009; Wójcikowska et al., 2013), *BABY BOOM* (*BBM*) (Boutilier et al., 2002), *WUSCHEL* (*WUS*) (Zuo et al., 2002), and *AGAMOUS-LIKE15* (*AGL15*) (Harding et al., 2003; Zheng et al., 2013).

In the regulation of the TF expression micro-RNA molecules (miRNAs), the products of *MIRNA* (*MIR*) genes have an essential function. miRNAs are single-stranded RNA molecules of 21–24 nucleotides that regulate the expression of the genes that are involved in plant development (for review, Bartel, 2009; Rubio-Somoza and Weigel, 2011). The biogenesis of mature miRNAs, which are the functional products of the *MIR* genes, is a multi-stage process that involves numerous interacting proteins. The primary *MIR* transcripts (pri-miRNA) are processed by DCL1 (DICER LIKE 1) RNase III, that is accompanied by the double-stranded RNA binding protein HYPONASTIC LEAVES 1 (HYL 1), the C2H2-zinc finger protein SERRATE (SE), and two cap binding proteins, CBP20 and CBP80/ABH1 (for review, Voinnet, 2009). In addition, the DDL (DAWDLE) protein was proposed to stabilize pri-miRNAs and facilitate the maturation of miRNA (Yu et al., 2008). As a result, the miRNA/miRNA^*^ duplex that is produced in the nucleus of a plant cell is transported to the cytoplasm where the miRNA strand is bound by the protein of the ARGONAUTE (AGO) family to form the RNA-Induced Silencing Complex (RISC) engaged in the recognition of the target transcripts that are complementary to the miRNA sequence (Baumberger and Baulcombe, 2005). Then, the miRNA-loaded RISC directs the post-transcriptional silencing of the targeted mRNA via its cleavage or translation repression (Tang et al., 2003; Brodersen et al., 2008).

The transcripts that are produced by members of the *MIR* gene family are processed to the identical or almost identical mature miRNA molecules. Different members of the *MIR* gene family are expressed in a developmental and tissue-specific manner and in response to various biotic and abiotic stimuli (Zhao et al., 2007, 2011; Moldovan et al., 2010; Kruszka et al., 2014).

Similar to the widely documented involvement of miRNA molecules in plant development *in vivo* (Jin et al., 2013), the expression of miRNAs was reported during *in vitro* induced SE in several plant species including *Citrus sinensis, Dimocarpus longan, Gossypium hirsutum, Larix kaempferi, Larix leptolepis, Liriodendron tulipifera*×*L. chinense, Manihot esculenta*, and *Zea mays* (Zhang et al., 2012, 2014; Li et al., 2013; Lin and Lai, 2013; Yang et al., 2013; Chávez-Hernández et al., 2015; Wu et al., 2015; Lin et al., 2015a,b; Khatabi et al., 2016). Thus, the engagement of miRNAs in the embryogenic transition that is induced *in vitro* is assumed, although knowledge about the function of the specific miRNA in SE induction is very limited.

In Arabidopsis, which is a model plant that has greatly contributed to the present knowledge on the genetic regulation of SE (Wójcikowska and Gaj, 2016), analysis of the *MIR*s/miRNAs that are associated with embryogenic induction has not yet been conducted. Thus, in the present study the expression profiles of 190 *MIR* genes that represented 114 *MIR* gene families was monitored during SE induction in an embryogenic culture of Arabidopsis. The analysis of the primary *MIR* transcripts was followed by the identification of mature miRNAs that were differentially accumulated during the embryogenic transition. A comparison of the pri-miRNA and the cognate mature miRNA level implied that an extensive differential processing of the primary *MIR* transcripts precedes the production of the functional miRNA molecules that are engaged in SE induction. The identified set of candidate miRNAs provides a valuable platform for further analysis that is aimed at deciphering the miRNA-mediated regulatory network that controls the embryogenic transition in plants.

## Results

### A vast number of *MIR* genes is transcribed during SE induction

**Figure 1:**
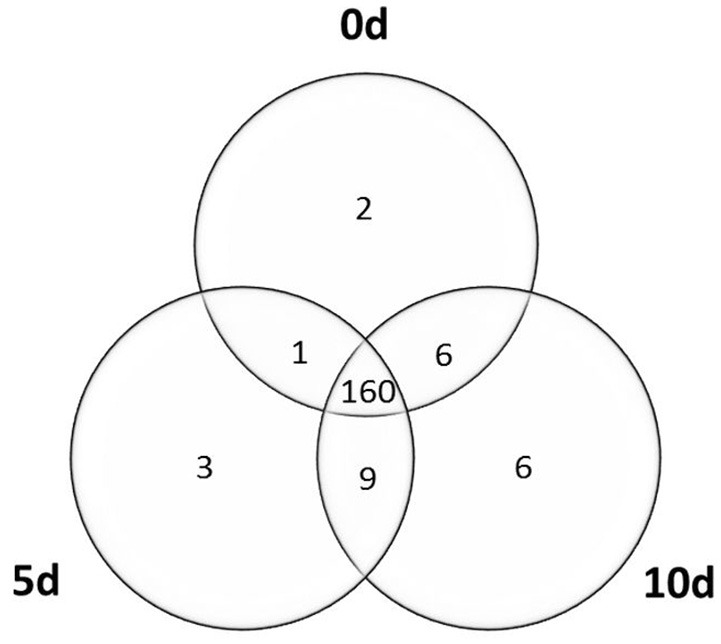
**Venn diagram of the ***MIRNA*** genes transcribed in the explants (0 d) and the derived embryogenic culture (5 and 10 d)**.

Our analysis indicated that a great majority (98%) of the analyzed *MIR* genes were expressed in the Col-0 explants and in the derived embryogenic culture. In total, primary transcripts of 187 *MIR* genes were detected at different time points of the culture (Figure 1; Table S1). A significant fraction (160; 86%) of the analyzed *MIR*s was transcriptionally active at all of the monitored time points (0, 5, and 10 d). SE induction resulted in the activation of a relatively low number (18) of *MIR* genes that were not expressed in freshly isolated explants (0 d) and a similar number (12–15) of the transcripts was induced at the early and the advanced stage of SE induction. We observed that the majority of the detected *MIR* transcripts (185; 99%) were expressed at both of the stages of the culture that were analyzed (5 and 10 d) and only three and six of the *MIR* genes were transcribed exclusively at early or advanced SE, respectively.

### SE induction is associated with the extensive modulation of *MIR* transcripts

**Figure 2:**
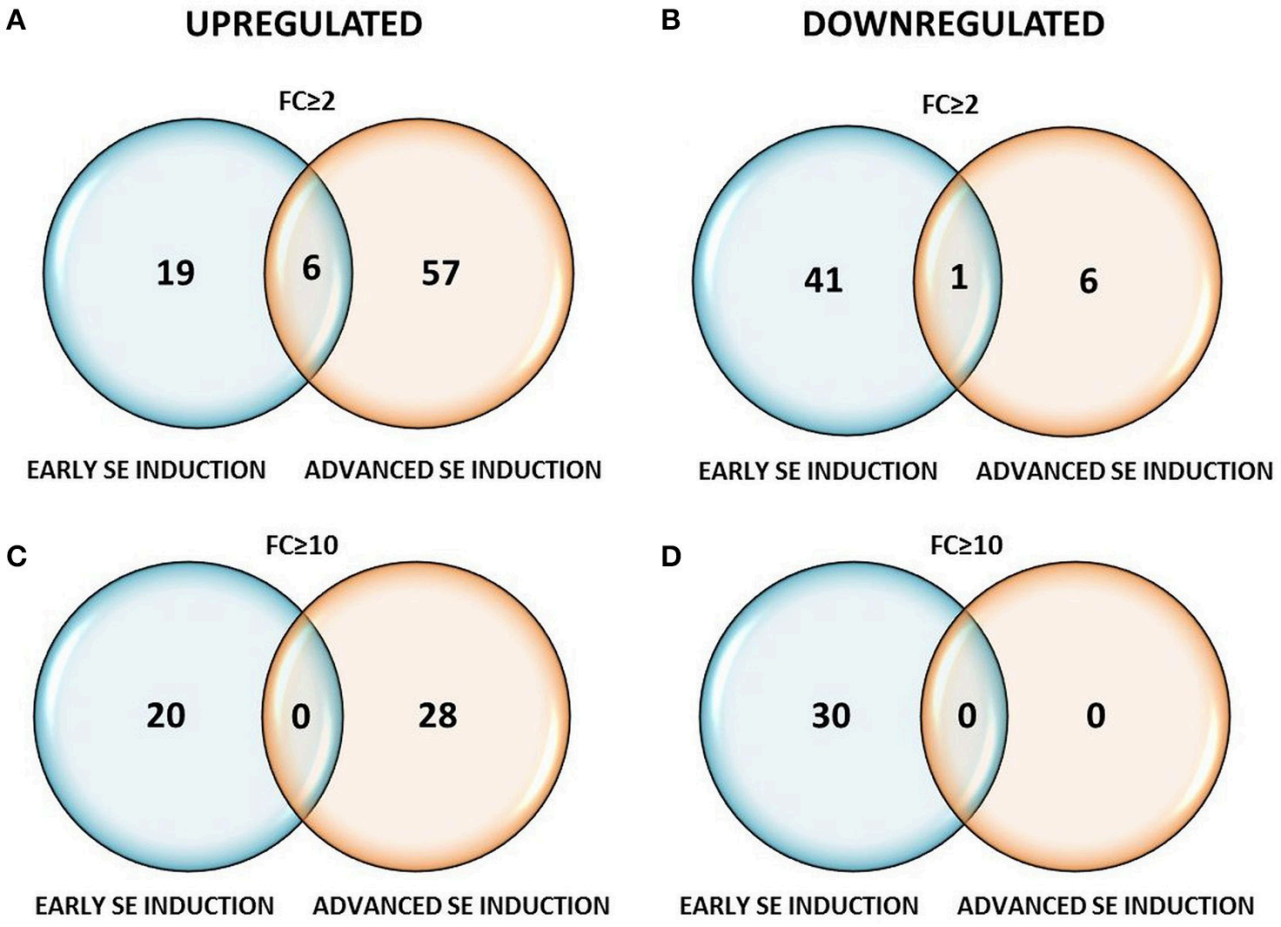
**Venn diagrams with the number of differentially expressed ***MIRNA*** genes at FC ≥ 2 (A,B)** and FC ≥ 10 **(C,D)** in the early (5–0 d) and advanced (10–5 d) stages of SE induction.

Profiling of the *MIR* genes expression revealed that the majority (120, 64%) of the transcripts displayed a significantly modulated expression (Fold change, FC ≥ 2.0) in response to SE induction (Table S2). A closer inspection of the *MIR* transcript expression across the SE culture indicated a similar number of differentially expressed *MIR* genes in the stages of SE induction that were compared: 67 (56%) vs. 70 (58%) of the *MIR* genes were found to be significantly modulated during the early (5–0 d) vs. the advanced (10–5 d) SE stage, respectively (Table 1). In contrast to the similar number of the modulated *MIR*s, the SE stages differed distinctly in the expression patterns of these genes. During early SE induction, a majority (42; 63%) of *MIR*s were significantly (FC ≥ 2.0) down-regulated and a large subset (30; 71%) of these genes was found to be highly repressed (FC ≥ 10.0) (Figure 2). In contrast to early SE induction, in advanced SE, the *MIR* genes were predominantly (63; 90%) up-regulated (FC ≥ 2.0) and almost half (28/63; 44%) of them displayed a highly stimulated transcription (FC ≥ 10.0).

**Table 1:** *****MIRNA*** genes differentially expressed in the early (5–0 d) and advanced (10–5 d) stages of SE induction**.

	**Number of differentially expressed genes**	**Down-regulated**	**Up-regulated**
**FOLD CHANGE X** ≥ **2**			
5–0 d	67 (56%)	42 (63%)	25 (37%)
10–0 d	79 (66%)	31 (39%)	48 (61%)
10–5 d	70 (59%)	7 (10%)	63 (90%)
**FOLD CHANGE X** ≥ **10**			
5–0 d	50 (75%)	30 (60%)	20 (40%)
10–0 d	30 (38%)	12 (40%)	18 (60%)
10–5 d	28 (40%)	0 (0%)	28 (100%)

**Figure 3:**
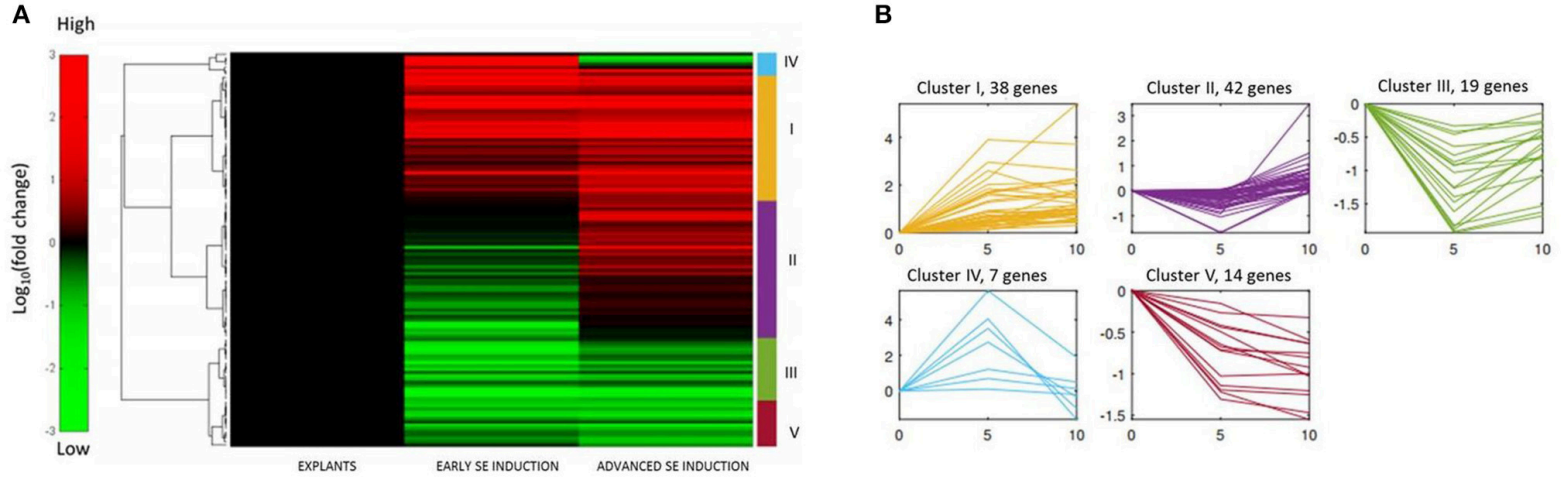
**Hierarchical clustering of the expression profiles of 120 ***MIRNA*** genes differentially expressed in SE culture. (A)** Heat map displaying the changes in the *MIR* expression in the early (5–0 d) and advanced (10–5 d) stages of SE induction, **(B)** Five patterns (I–V) of gene expression and the relevant number of *MIR* genes.

A set of 120 *MIR* genes that had a significantly modulated expression was subjected to hierarchical clustering and five distinct gene expression patterns were observed (Figure 3; Table S3). The analysis showed that numerous (61) genes that had been down-regulated at early SE were up-regulated at advanced SE induction (clusters II and III). In majority of these genes (42; 69%), the expression level at 10 d was found to be similar to 0 d of the culture (cluster II), which suggests a transient modulation of these genes. The opposite expression patterns, i.e., up-regulation in early SE followed by down-regulation in advanced SE, were noticed for a small number (7) of genes (cluster IV). Hierarchical clustering analysis also indicated numerous genes (38) that were consistently up-regulated during both of the monitored SE stages (cluster I) and a limited number of genes (14) were consistently down-regulated at both of the culture stages (cluster V).

In conclusion, numerous *MIR* genes were found to be differentially expressed during SE induction, and, for the majority of these genes, a distinct down-regulation at the early culture was followed by their up-regulation in the advanced culture.

The analyzed transcripts represented 114 *MIR* gene families of different sizes that ranged from 1 to 14 member genes. A closer inspection of the pri-miRNAs that were produced within the gene family indicated profound differences in the expression level in SE of the gene family members. Divergent transcript profiles of the member genes were found within the majority (79%) of the analyzed *MIR* families. Thus, a diverse contribution of the *MIR* genes of the same family to the regulation of SE induction is assumed.

### Mature miRNAs of SE-modulated accumulation level

To evaluate the regulatory impact of *MIR* genes on SE induction, the accumulation of the mature miRNAs that constitute the functional products of *MIR* transcripts was examined during the time course of SE. The mature miRNAs that were selected for the analysis represented the *MIR* genes that were differentially expressed in SE. A total of 19 mature miRNA molecules, which represented 60 pri-miRNAs encoded by 14 *MIR* gene families (*MIR156, MIR157, MIR159, MIR160, MIR164, MIR166, MIR168, MIR169, MIR172, MIR319, MIR390, MIR393, MIR396*, and *MIR398*), were subjected to stem-loop RT-qPCR analysis.

It was found that a great majority (85%) of the mature miRNAs that were analyzed (miR156a–f, miR156g, miR156h, miR157, miR159, miR160, miR164, miR166, miR169a–c, miR169d–g, miR169h–n, miR319a–b, miR319c, miR390, miR393, miR396, and miR398) were differentially accumulated during SE induction (Table S4). Two mature miRNAs, miR168 and miR172 that are encoded by the multigene *MIR* families, displayed a steady expression although the level of their pri-miRNA precursors was modulated in SE.

It was observed that the expression profiles of mature miRNAs differed distinctly in the early and advanced stage of SE induction. In general, the expression profile of the mature miRNAs that were analyzed corresponded with the global transcription pattern of the *MIR* genes observed during SE induction. Accordingly, in early SE the majority (69%) of mature miRNAs was found to be down-regulated whereas in the advanced SE stage, most of them were up-regulated (11; 79%) (Table 2). Corresponding expression profiles of pri- and mature miRNA were found for *MIR157a–c* and *MIR396a* in early SE and for *MIR169c, MIR169d, MIR169g, MIR319b*, and *MIR396b* in advanced SE. In contrast, some inconsistencies in the expression of pri- and mature miRNA were found for *MIR157d* and *MIR396b* in early and for *MIR169a, MIR169b, MIR319a*, and *MIR396a* in advanced SE.

**Table 2:** **Number of differentially expressed mature miRNAs in the early (5–0 d) and advanced (10–5 d) stages of SE induction**.

**Stage of SE induction**	**Number (%) of differentially expressed miRNAs**	**Down-regulated**	**Up-regulated**
Early	13 (68%)	9 (69%)	4 (31%)
Advanced	14 (74%)	3 (21%)	11 (79%)

**Figure 4:**
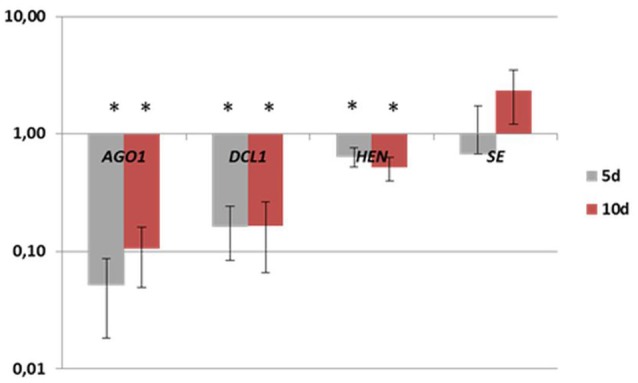
**Expression levels of ***AGO1***, ***DCL1***, ***HEN***, and ***SE*** genes in the early (5 d) and the advanced stage (10 d) of SE induction**. A value significantly different from 0 d is indicated with an asterisk (*p* ≤ 0.05).

A closer look into the expression of individual members of the *MIR* families indicated that only a small subset of the family transcripts seem to contribute to the production of mature miRNAs (Figure S1; Table 3). The *MIR* genes that had a rather dissimilar contribution to the production of the mature miRNAs were found within the majority (11; 79%) of the *MIR* gene families. The majority (38; 63%) of the analyzed *MIR* genes was transcribed at a significantly higher level than the relevant mature miRNAs, thus suggesting that numerous pri-miRNAs were not processed into the functional miRNA molecules. In support of this assumption we observed a significantly decreased level of the transcripts that encode the key enzymes of the pri-miRNA processing machinery including *DCL1* and *HEN1* (Figure 4; Table S5).

**Table 3:** **Accumulation (up-regulation in red and down-regulation in gray) of the primary ***MIRNA*** transcripts (pri-miRNA) and the relevant mature miRNAs in early (5–0 d) and advanced (10–5 d) SE induction**.

*****MIRNA*** gene**	**FC**		**mature miRNA**	**FC**	
	**SE INDUCTION**			**SE INDUCTION**	
	**EARLY**	**ADVANCED**		**EARLY**	**ADVANCED**
156a	0.36^*^	0.69^*^			
156b	3.48	3.39	156a–f	0.06^*^	0.8
156c	0.56	4.67^*^			
156d	0.27	25.86^*^			
156e	0.77	15.07^*^			
156f	0.32^*^	0.96			
156g	0.02^*^	42.11^*^	156g	0.14	0.79
156h	0.05^*^	0.82	156h	0.04^*^	15.84
157a	50.28^*^	3.89^*^	157	1.93	26.53^*^
157b	51.68^*^	3.84^*^			
157c	45.09^*^	0.5^*^			
157d	0.01^*^	5.93^*^			
159a	0.53^*^	2.9^*^	159	0.07^*^	2.1^*^
159b	2.82	4.76^*^			
160a	0.75	4.14^*^	160	0.51^*^	0.04^*^
160b	0.23^*^	3.37^*^			
160c	2.46	3.59^*^			
164a	0.24^*^	8.22^*^	164	0.04^*^	3.32
164b	0.43^*^	2.76^*^			
164c	0.12^*^	4.08^*^			
166a	0.19^*^	0.9	166	0.12^*^	0.3^*^
166b	1.17	0.57^*^			
166c	0.18	90.81^*^			
166d	3.36	0.63			
166e	0.09^*^	13.83^*^			
166f	7.25	1.64^*^			
166g	1.21	1.5^*^			
168a	6.74^*^	1.31	168	1.2	1.41
168b	0.19^*^	14.36^*^			
169a	4.91^*^	1.47	169a–c	0.57	13.47^*^
169b	24.78	0.98			
169c	0.11	32.65			
169d	122.01	25.13			
169e	131.59	1.26			
169f	154.13	0.96	169d–g	0.94	26.5
169g	3.25	2.79			
169h	1.55^*^	1.64^*^			
169i	0.05	1.64^*^			
169j	9.74	0.72	169h–n	2.9^*^	2.6^*^
169k	2.7	1.05			
169l	6.19	0.97			
169m	3.79	0.57^*^			
169n	907.92^*^	0.95			
172a	0.96	6.66	172	0.54	1.78
172b	2.48^*^	2.29^*^			
172c	70.97^*^	0.41^*^			
172d	21.79^*^	2.80^*^			
172e	0.38^*^	3.48^*^			
319a	8.46^*^	1.84	319a–b	0.17^*^	3.97^*^
319b	2.79^*^	2.31^*^			
319c	1.81	4.97^*^	319c	0.17^*^	4.04^*^
	47.72	1.77	390	2.54^*^	0.79
390a	5.49^*^	0.84			
390b					
393a	37.6^*^	0.77^*^	393	12.41^*^	0.04^*^
393b	18.87^*^	2.5			
396a	105.81^*^	1.21	396	2.82	5.12^*^
396b	0.03^*^	3.48			
398a	0.91	3.53	398	0.07^*^	36.67^*^
398b	0.14^*^	5.65^*^			
398c	0.02^*^	49.75^*^			

FC, Fold change;

* *significant difference between compared days of SE culture (p ≤ 0.05)*.

In some instances, a delayed accumulation of mature miRNA was found in respect to the expression of the relevant pri-miRNA. Accordingly, the increased expression of *MIR169h–n* in early SE was followed by a large accumulation of mature-miRNA in advanced SE. A gradual accumulation of these molecules in the tissues undergoing SE induction cannot be excluded when attempting to explain a delayed increase of the mature miRNA.

In conclusion, in contrast to the robustly modulated transcription of the *MIR* genes accumulation of the mature miRNAs seem to be rather confined during embryogenic response. Hence, the extensive post-transcriptional regulation is assumed to be associated with the production of the functional miRNAs that control the reprogramming of the somatic cells into embryogenic cells.

### Functional annotation of the candidate miRNAs

Mature miRNA molecules with a differential accumulation in SE induction (miR156/miR157, miR159, miR160, miR164, miR166, miR169, miR319, miR390, miR393 miR396, and miR398) were selected as the candidate regulators of the embryogenic response. In support of the involvement of the candidate miRNA in the regulation of SE, all of these molecules have been reported to control hormone and/or stress responses. Moreover, among the candidate miRNAs, those that had a documented impact on the development of zygotic embryos (miR156/157, miR164, miR166, and miR169), seeds (miR159), leaves (miR159, miR164, mir319, miR390, and miR396), roots (miR160, miR169, miR390, miR393, and miR396), and flowers (miR159, miR164), as well as the control of flowering (miR156/miR157, miR159, and miR169) and vegetative phase transition (miR156/157, miR169, and miR390) were found (Table S6).

**Figure 5:**
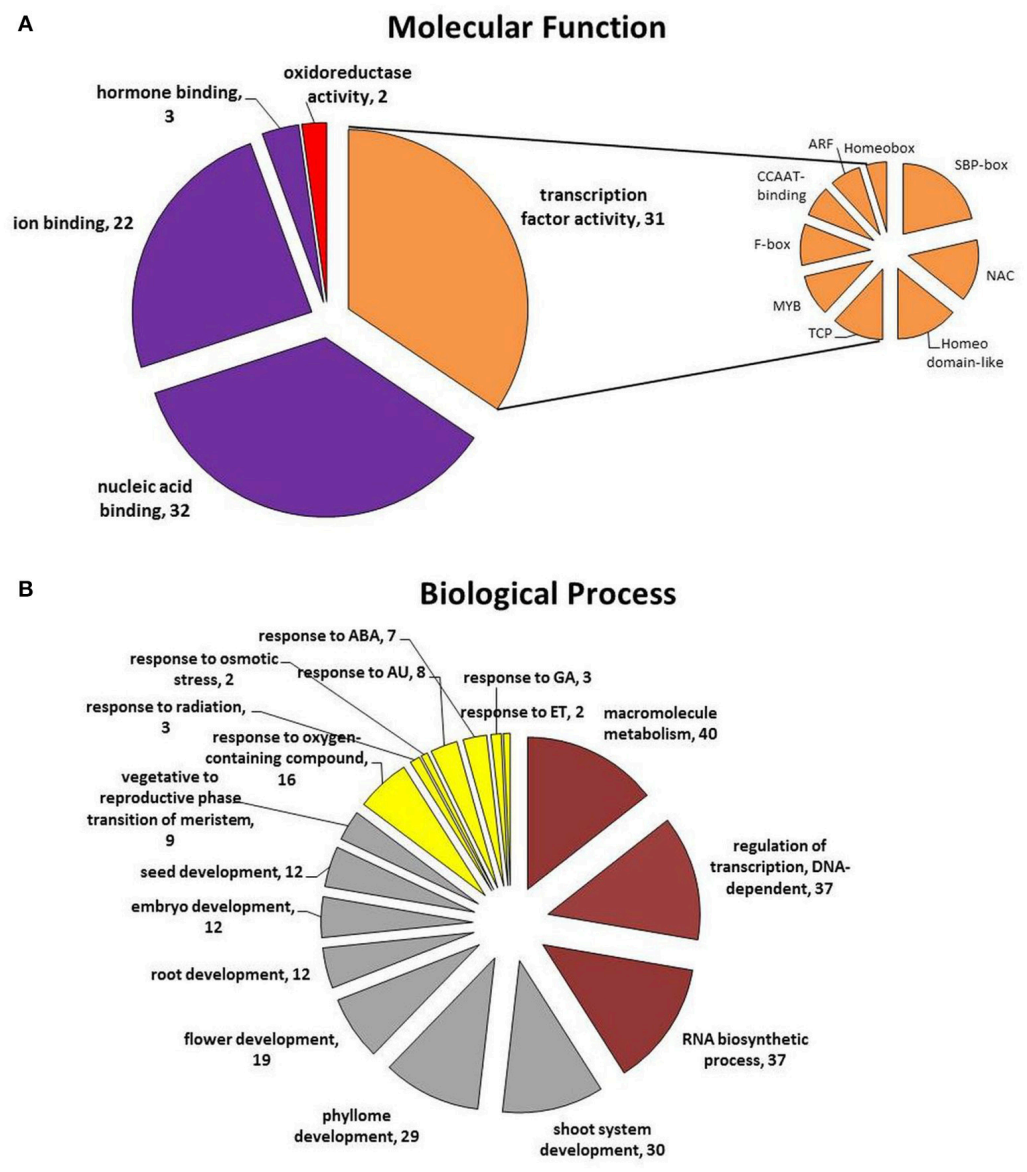
**Functional categories: (A)** molecular function and **(B)** biological process of the target genes annotated to the candidate miRNAs that are assumed to be involved in SE induction. ABA, abscisic acid; AU, auxin; ET, ethylene; GA, gibberellic acid.

To further assess the potential pathways that are controlled by the candidate miRNAs during SE induction, the functions of their predicted targets were annotated (Table S7). A total of 59 target genes were subjected to Gene Ontology (GO) analysis and 52 of them were significantly (at *p* < 0.01) enriched for 235 GO terms over two main functional categories–molecular function (MF) (16) and biological processes (BP) (219) (Figure 5; Tables S8, S9). The highly enriched GO terms in the MF category are related to transcription factors (TF) (GO:0001071; 31 genes); binding (GO:0005488; 48 genes) including nucleic acids (32 genes), ions (22 genes), and hormones (3 genes), and oxidoreductase catalytic activity (GO:0016721; 2 genes) (Figure 5A). TFs targeted by the candidate miRNA were annotated to nine gene families that are referred to as SBP-box (9), NAC (6), Homeodomain-like (6), TCP (5), MYB (4), F-box (4), CCAAT-binding (3), ARF (3), and Homeobox (2).

The annotated to the candidate miRNA GO terms infer that a wide range of biological processes is involved in regulation of SE induction (Figure 5B). The main, highly enriched BP categories are related to metabolic (GO:0008152; 50 genes) and developmental (GO:0032502; 39 genes) processes and the responses to stimuli (GO:0051716; 19 genes) (Table S9).

Among the targets of the candidate miRNAs, those that are related to metabolic processes were found to be overrepresented in the BP category. Within this category, numerous target genes that are involved in the regulation of the macromolecule metabolic process (GO:0060255), the regulation of transcription (GO:0006355) and RNA biosynthesis (GO:2001141) were identified.

Targets of the great majority (92%) of the candidate miRNAs were annotated to plant development, a general category that covers diverse developmental processes including phyllome (GO:0048827), shoot (GO:0048367), and root development (GO:0048364), the transition of the meristem from the vegetative to the reproductive phase (GO:0010228) and the regulation of the development of the reproductive structures (GO:0048608), such as flowers (GO:0009908), embryos (GO:0009793), and seeds (GO:0048316). Among the genes that are related to zygotic embryogenesis, which is a process that corresponds to somatic embryo development, the targets of miR164, miR166, miR169, miR319, and miR396 were identified.

Importantly to the mechanism of SE induction, another functional category that was highly enriched in genes was found to be related to the response to stimuli (GO:0051716) and targets of 92% of the candidate miRNA were annotated to this functional category, including numerous genes that are involved in plant responses to hormones, particularly auxin (GO:0009733), abscisic acid (GO:0009737), ethylene (GO:0009723), and gibberellic acid (GO:0071370), osmotic stress (GO:0071470) and radiation (GO:0071478). The genes that are related to hormone signaling were identified within the targets of miR159, miR160, miR164, miR319, miR393, and miR396.

### Verification of the function of the candidate miRNAs in SE–target analysis

**Figure 6:**
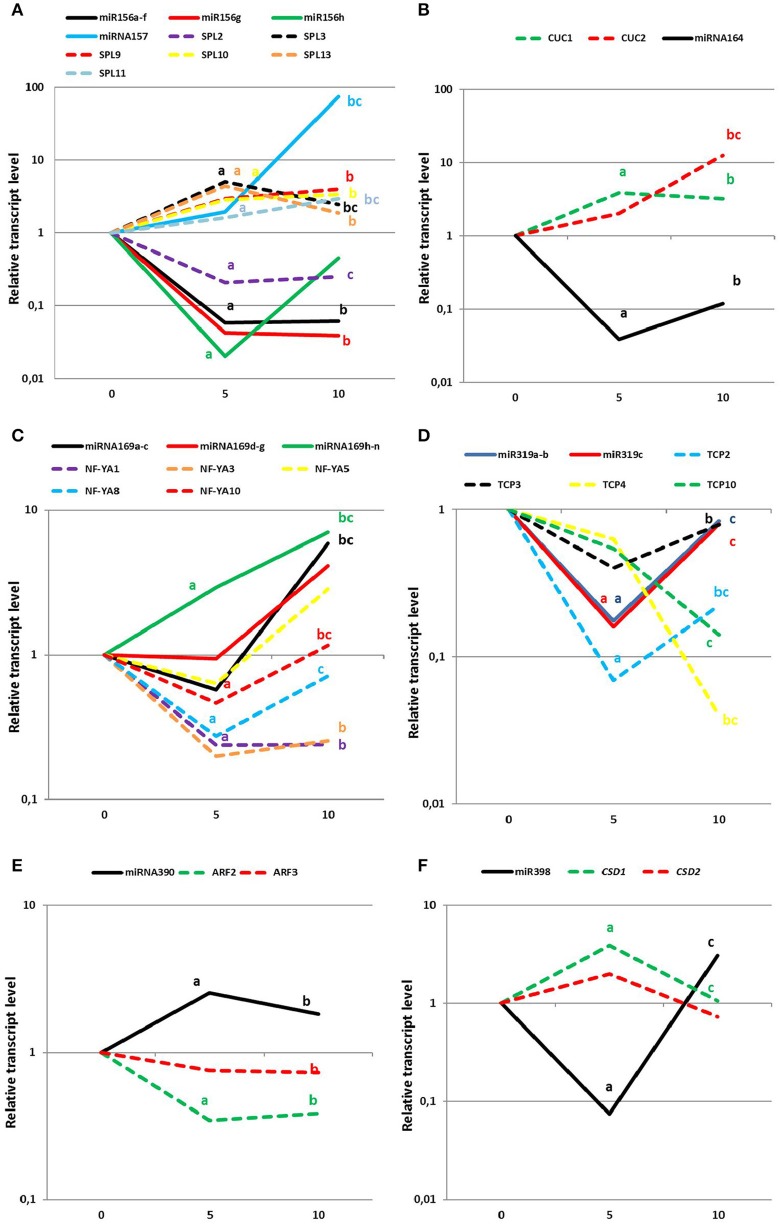
**Expression levels of mature miRNAs (solid lines) and the relevant target genes (dotted lines) at day 0, 5, and 10 of SE induction. (A)** miR156/157, **(B)** miR164, **(C)** miR169, **(D)** miR319, **(E)** miR390, and **(F)** miR398. Lower case letters indicate a significant difference between 5 and 0 d (a); 10–0 d (b); and 10–5 d (c) (*p* ≤ 0.05).

To confirm the involvement of the candidate miRNAs in SE, the expression patterns of the presumed miRNA targets were analyzed in an embryogenic culture. In total, the expression levels of 21 genes that are targeted by seven candidate miRNAs were examined (Figure 6; Table S5). An analysis of seven *SPL* (*SPL2,3,9,10,11,12*, and *13*) genes, which are possible targets of miR156/miR157, revealed that all of them displayed a significant accumulation of the transcripts during SE except for *SPL2*. The *SPL*s with an up-regulated expression (*SPL3,9,10,11,1*, and *13*) may be controlled by miR156 due to the decreased expression of this miRNA during SE. In addition, the results of the expression profiling infer a regulatory interaction of *SPL2* and miR157 in SE due to the up-regulation of miR157 and down-regulation of *SPL2* that was observed in embryogenic culture (Figure 6A).

The elevated accumulation of both miR164 targets, *CUC1* and *CUC2* genes, throughout SE induction was found to be opposite to the down-regulation of miR164, thus suggesting that an miR164-*CUC* regulatory node might operate in SE induction (Figure 6B). Within the miR169 targets, three genes of *NF-YA* family, *NF-YA1, NF-YA8* and *NF-YA10*, displayed a significant decrease in the early stage of SE induction, which contrasted with the strongly increased level of miR169h–n. These results suggest that a biological function of miR169 in SE seems to be related to the repression of *NF-YA* genes (Figure 6C). In SE, a regulatory relation between miR319 and the genes of the *TCP* family cannot be excluded as the substantially increased level of miR319 that was observed in the advanced stage of the embryogenic culture was found to be accompanied by a significantly decreased expression of the *TCP4* and *TCP10* transcripts (Figure 6D).

The expression profiling indicated that among the *TF* genes that are regulated by the candidate miRNAs, key regulators of the auxin response also need to be considered. Accordingly, miR390 might control SE induction via contribution to production of the tasiARFs that repress *ARF2, ARF3*, and *ARF4* transcripts. In support for this assumption a distinct down-regulation of *ARF2* and *ARF3* transcripts was observed in the early and advanced embryogenic culture coupled with a significant accumulation of miR390 during SE (Figure 6E). The target analysis indicated that besides the regulation of the genes that encode TFs, the candidate miRNA might also control the key enzymes that are involved in a stress response. In support of this assumption, the *CSD1* gene seems to be controlled by miR398 during both stages of SE because inverse expression patterns of the target transcripts vs. mature miRNA molecules was observed (Figure 6F).

## Materials and methods

### Experimental design

The expression profiles of 190 *MIR* genes belonging to 114 gene families were monitored at the level of the primary *MIR* transcripts using mirEX, a high throughput RT-qPCR platform (Bielewicz et al., 2012; Zielezinski et al., 2015, http://comgen.pl/mirex2/). The analysis encompassed the tissue at different stages of embryogenic culture derived from immature zygotic embryo (IZE) explants that had been induced on an auxin medium. The experimental design followed the analysis of SE-associated TF transcripts (Gliwicka et al., 2013) and included freshly isolated explants (0 d) and the embryogenic induction at the early (5 d) and advanced (10 d) stages. To identify the *MIR* genes that were differentially expressed during SE, the pri-miRNA transcript levels at 5 vs. 0 d, 10 vs. 0 d, and 10 vs. 5 d were analyzed. The level of mature miRNAs products of the selected *MIR* that displayed a significantly modulated expression was evaluated using stem-loop RT-qPCR.

### Plant material

The *Arabidopsis thaliana* (L.) Heynh. Col-0 genotype was analyzed in this study. Seeds of Col-0 were supplied by NASC (The Nottingham Arabidopsis Stock Centre). Plants were grown in soil pots in a “walk-in” type phytotron under controlled conditions (20–22°C, 16/8 h L/D photoperiod, light intensity of 100 μE/m^2^s).

### Somatic embryogenesis induced *in vitro*

Somatic embryogenesis was induced following the standard protocol (Gaj, 2001). IZE at the mid-late cotyledonary stage of development (10–12 days after pollination) were used as explants. The SE induction medium (E5) was based on B5 basal micro- and macro-salts (Gamborg et al., 1976) and supplemented with 20 g/L sucrose, 8 g/L Oxoid agar, and 5 μM 2,4-D. Cultures were maintained in a growth chamber under controlled conditions: 22°C, 16/8 h (light/dark), light intensity 50 mE/m^2^s.

### RNA isolation and cDNA synthesis for the analysis of *MIR*s

SE cultures were sampled at three time points for the isolation of total RNA: freshly isolated IZEs (0 d) and explants at the early (5 d) and the late (10 d) stage of SE induction. At each time point, three biological replicates were used for the analysis.

To analyse the transcripts of *MIR* genes (pri-miRNA), total RNA was isolated using an RNAqueous Kit (Ambion by Life Technologies) according to the manufacturer's instructions. The RNA concentration was measured using Nano-Drop ND-1000 (NanoDrop Technologies, Wilmington, Delaware, USA) and RNA integrity was tested on 1% agarose gel. Reverse transcriptase reactions were performed using 3 μg of RNA, Oligo(dT)20 Primer (Invitrogen by Life Technologies) and SuperScript III Reverse Transcriptase (Invitrogen by Life Technologies) according to the manufacturers' instructions.

### RNA isolation and cDNA synthesis for mature miRNAs analysis

To evaluate the accumulation level of mature miRNAs, total RNA was isolated using a miRVana Kit (Ambion by Life Technologies) according to the manufacturer's instructions. The design of the oligonucleotides and stem-loop reverse transcriptase reactions were performed according to Speth and Laubinger (2014).

### RNA isolation and cDNA synthesis for the analysis of target genes

Total RNA was isolated using an RNAqueous Kit (Ambion by Life Technologies) according to the manufacturer's instructions. Reverse transcriptase reactions were performed using a RevertAid First Strand cDNA Synthesis Kit (Fermentas) according to the manufacturer's instructions.

### Quantitative PCR for profiling the *MIR*s

To monitor the accumulation of pri-miRs during SE, the RT-qPCR reaction was used. Gene-specific primers for 190 microRNAs from *A. thaliana* genes were designed as described in Szarzynska et al. (2009) and Bielewicz et al. (2012). The primer sequences are available on the miREX website (http://www.comgen.pl/mirex1/). RT-qPCR reactions were performed using a 7900HT Fast Real-Time PCR System (Applied Biosystems by Life Technologies) and PowerSYBR Green to monitor the dsDNA synthesis. The reaction mixture (10 μL) contained 5 μL of 2x PowerSYBR Green PCR Master Mix (Applied Biosystems by Life Technologies), cDNA and gene-specific primers (200 nM each). The following thermal profile was used for all of the qPCRs: 95°C for 10 min; 40 cycles of 95°C for 15 s; and 60°C for 1 min. After each RT-qPCR run, dissociation curve analyses were performed. The results were analyzed using SDS 2.2.1 software (Applied Biosystems by Life Technologies) (Szarzynska et al., 2009). Ct values for all of the *MIR* transcripts were normalized to the *PP2AA3* (*AT1G13320*) and *ELONGATION FACTOR 1-ALFA (EF-1)* (*AT1G07930*) (Czechowski et al., 2005).

### Quantitative PCR for profiling mature mirnas

qPCR analyses were performed using a LightCycler 480 (Roche) to monitor the accumulation of mature miRNAs. The following RT-qPCR reaction conditions were used: Denaturation one repeat of 10 min at 95°C followed by 45 repeats of 10 s at 95°C, 8 s at the specific temperature for each of the primers, 12 s at 72°C and 5 s at 80°C. Denaturation for the melt curve analysis was conducted at 95°C followed by 15 s at 65°C and 95°C (0.1°C/s for fluorescence measurement). The stem-loop primers for the reverse transcription and the primers for RT-qPCR are listed in Table S10. The universal qPCR reverse primer sequence was designed according to Wu et al. (2007). Ct values were normalized to the *EF-1* (*AT1G07930*).

Expression data were submitted to the mirEX qPCR platform (Zielezinski et al., 2015; http://comgen.pl/mirex2/).

### Quantitative PCR for profiling the target genes

qPCR analyses were performed using a LightCycler 480 (Roche) to monitor the accumulation of target gene transcripts. The following RT-qPCR reaction conditions were used: Denaturation–one repeat of 10 min at 95°C, followed by 45 repeats of 10 s at 95°C, 8 s at the temperature that is specific for the primer pairs, 12 s at 72°C and 5 s at 80°C. Denaturation for the melt curve analysis was conducted at 95°C followed by 15 s at 65°C and 95°C (0.1°C/s for the fluorescence measurement). Ct values were normalized to the *EF-1* (*AT1G07930*). The primers used for the RT-qPCR reactions are listed in Table S11.

### Gene and mature miRNA expression level analysis

The fold change (FC) of the SE-modulated *MIRs*, mature miRNAs and their presumed targets was calculated using the comparative 2^(−Δ*ΔCt*)^ method. In all of the analyzed culture tissue samples, the control genes displayed a constant expression pattern with Ct = 17 ± 1 and Ct = 18 ± 1 for *PP2AA3* and *EF-1*, respectively. Candidate genes were identified using the thresholds of 2- and 10-fold changes. The FC were calculated as the ratio of the transcript levels at different SE time-points (SE-modulated genes). The reactions and calculations were performed in biological triplicate. *P*-value was calculated by comparisons of dCt values. As the number of data was limited it was not possible to conduct normality tests, thus non-parametric Analysis of Variance (Kruskal Wallis test) was applied to calculate the significant differences between the comparisons (corrected *p* ≤ 0.05).

### Hierarchical clustering of SE-modulated *MIR*s

The expression profiles were clustered hierarchically using the average linkage method and Euclidean distances. The experiment was repeated for the number of clusters k = 2, 3, …, 15. For each k, the results were visualized and manually verified. A number of clusters, k = 5, was selected for further analysis as the one that was characterized by the highest within-cluster coherence and between-cluster separation. The clustering procedure and the visualizations were performed using MATLAB R2014a software.

### Target prediction and functional annotation

The miRNA targets that were predicted using the psRNATarget tool (Dai and Zhao, 2011) were functionally annotated with using the PLAZA Dicots database version 3.0 (http://bioinformatics.psb.ugent.be/plaza/versions/plaza_v3_dicots/ots/). The significance of the over-representation was determined using the hypergeometric distribution followed by the Bonferroni method for multiple testing corrections (corrected *p* ≤ 0.01).

## Discussion

### Extensive and specific to SE-stage modulation of *MIRNA* genes is associated with the embryogenic transition induced in arabidopsis

The study indicated that numerous *MIR* genes are active in the embryogenically induced somatic tissue of Arabidopsis and that the majority of them are significantly modulated during SE induction. Interestingly, the expression level of *MIR*s seems to be specific to the stage of SE induction and a substantial repression vs. stimulation of *MIR* genes was found to be characteristic to the early vs. advanced stage of SE induction, respectively. Like the *MIR* transcripts, the majority of mature miRNAs were found to be down-regulated during early SE and up-regulated in the advanced SE induction stage. Inverse transcription profile i.e., substantial transcript accumulation in the early SE followed by transcript down-regulation in the advanced SE stage, displayed *TF* genes expressed in embryogenic culture of Arabidopsis (Gliwicka et al., 2013). This observation was a reason to focus on TFs as miRNA targets. Thus, it seems that in SE, similar to ZE, miRNAs might contribute to the cellular differentiation during embryonic development via the regulation of the *TF* genes (Nodine and Bartel, 2010). In support of this assumption, we found genes encoding TFs to be over-represented among the targets that were annotated to the differentially expressed miRNAs. Thus, the intense miRNA-mediated regulation of *TF* genes could be employed in the mechanism of SE induction, similar to other developmental processes (Chen and Rajewsky, 2007; reviewed in Hobert, 2008). Moreover, a regulatory feedback loop between the TF and *MIR* genes might be expected during SE considering that binding sites for various TFs were identified within the *MIR* promoters (Megraw et al., 2006). In support of this assumption, the *GRF1* (*GROWTH RESPONSE FACTOR1*) and *GRF3*, which are the targets of miR396 that were found to be differentially expressed in SE, were reported to repress the expression of *MIR396a* and *MIR396b* in Arabidopsis (Hewezi and Baum, 2012). Likewise, ARFs (AUXIN RESPONSE FACTOR) might control the SE-modulated expression of *MIR160, MIR167*, and *MIR390* due to the AUXIN RESPONSE ELEMENTS (AREs) that were detected in the promoters of these genes (Gutierrez et al., 2009; Yoon et al., 2009; Marin et al., 2010).

In conclusion, the study provides comprehensive evidence that the regulatory interactions between TFs and miRNA play a pivotal role in the re-programming of somatic cells into embryogenic cells.

### Intense post-transcriptional regulation of miRNA is associated to SE induction

The present comparative analysis of pri- and mature-miRNAs levels in an embryogenic culture of Arabidopsis indicated a global similarity in the expression profiles of these molecules. However, the up-regulation of the individual pri-miRNAs that were produced within a gene family did not always result in the accumulation of the functional product, i.e., mature miRNA. A growing number of reports have highlighted the importance of the post-transcriptional regulation of miRNA biogenesis in plant and animal development (Lee et al., 2008; Nogueira et al., 2009; Bielewicz et al., 2013; Barciszewska-Pacak et al., 2015). The extensive differential processing of the primary miRNA transcripts that was inferred in the present study appears to reflect the response of the cultured tissue to the stress conditions that were applied *in vitro* to induce SE. Various environmental stresses have been shown to trigger the differential processing of the primary miRNA transcripts, which is relevant to this assumption (Yan et al., 2012; Jia and Rock, 2013). A fundamental role of post-transcriptional regulation of miRNA expression in the responses to various abiotic stresses was recently postulated in Arabidopsis seedlings and, similar to the present results, a broad modulation of pri-miRNA was found to distinctly contrast to the rather confined response of mature miRNA (Barciszewska-Pacak et al., 2015).

Among the various mechanisms that control the biogenesis of miRNA, splicing efficiency, alternative splicing and polyA site selection have been postulated to be frequent (Bielewicz et al., 2013; Szweykowska-Kulińska et al., 2013). Alternative splicing may result in a low level of mature miRNA in spite of the up-regulation of the relevant pri-miRNA as was indicated for miR400 and miR846 under heat stress and ABA treatment, respectively (Yan et al., 2012; Jia and Rock, 2013). The discrepancy between the accumulation of pri- and mature miRNA might result from the differential susceptibility of pre-miRNA to the processing machinery and the diverse stability of mature miRNA (Ramachandran and Chen, 2008; Köster et al., 2014; Dolata et al., 2016). Recently, the increased levels of miRNA161 and miRNA173 coupled with down-regulated expression of the relevant pri-miRNAs were described in Arabidopsis seedlings subjected to salinity stress and ARGONAUTE 1 (AGO1) was proposed to be involved in the co-transcriptional regulation of *MIR* gene expression (Dolata et al., 2016).

Among the post-transcriptional processes that control the accumulation of mature miRNA, the level of the proteins that are involved in microRNA biogenesis has been postulated to regulate the efficiency of pri-miRNA processing into mature miRNAs (Rogers and Chen, 2013; Wang et al., 2013). In accordance with this postulate, in the present study, a lower than expected accumulation of the functional miRNA products in SE was coupled with the down-regulation of the genes encoding key enzymes in miRNA processing (*AGO1, DCL1, HEN1*, and *SE*).

The differential stability of miRNAs has mostly been demonstrated in animal cells and the active degradation of mature miRNAs has been identified as an important mechanism in miRNA homeostasis (Bail et al., 2010; Rüegger and Großhans, 2012). Thus, it cannot be ruled out that the high degree of stability of miRNA may account for the delayed accumulation (in the advance SE stage) of the mature products of the *MIR169h–n* genes of up-regulated transcription in the early stage of SE.

An inconsistency in the expression of pri- and mature miRNA was also observed for miR168. A steady level of mature miR168 was detected throughout SE in contrast to the differential expression of both members of the family (*MIR168a* and *MIR168b*). miR168 was found to control miRNA processing by targeting a key gene in this pathway, *AGO1* (Vaucheret et al., 2004, 2006). However, in SE, other regulatory elements besides miR168 seem to control *AGO1* as we observed a down-regulated transcription of the *AGO1* transcripts in the culture that had a steady miR168 level. In support of this suggestion, complex regulatory loops were postulated to be involved in the control of *AGO1* in order to ensure the correct function of the miRNA and siRNA pathways (Mallory and Vaucheret, 2009).

The results show that in the majority (79%) of the analyzed *MIR* gene families, the members displayed distinctly divergent expression profiles during SE. Thus, the diverse contribution of different members of the *MIR* family to the production of mature miRNAs, and hence, the regulation of SE is assumed. In support of this supposition, the functional diversification within *MIR* gene families was indicated in Arabidopsis. Accordingly, it was shown that *MIR393a* contributes to bacterial resistance (Navarro et al., 2006), *MIR164a* controls leaf differentiation (Koyama et al., 2010) and the expression of *MIR165a/MIR166a* is specific to the abaxial epidermis (Yao et al., 2009).

### Stress- and hormone-related functions of the candidate miRNAs during SE induction

Stress factors together with hormone treatments are widely accepted to play a pivotal role in the mechanism of SE induction (Jiménez, 2005; Zavattieri et al., 2010). In support of this belief, the promoters of *MIR* genes, including those that are differentially expressed in the SE of Arabidopsis (present study), have been found to be highly enriched in the *cis* regulatory elements that control stress- and hormone-responses (Megraw et al., 2006; Zhao and Li, 2013).

The miRNAs that have a differential accumulation in SE that were reported to control plant responses to stress include **miR398**. It can be assumed that miR398 contributes to SE induction via the activation of a stress protective reaction (Sunkar et al., 2006). In support of this postulate, we observed that the down-regulated expression of miR398 in early SE was accompanied by a significant up-regulation of the *CSD1* (Cu/Zn superoxide dismutase 1) gene encoding a key enzyme that is involved in the responses to oxidative stress (Sunkar et al., 2006). The decreased accumulation of **miR398** linked with an increased transcription of the *CSD* genes was also indicated in embryogenic cultures of other plants (Zhang et al., 2012; Lin and Lai, 2013). Another stress-related candidate for a possible regulatory role in SE, **miR169**, was reported to be highly produced in response to different stresses in Arabidopsis, tomato and rice (Zhao et al., 2009, 2011; Zhang et al., 2011). The present results suggest that during SE, miR169 might target the *NF-YA* (*NF-YA1, NF-YA8*, and *NF-YA10)* genes encoding the HAP2-type transcription factors, which are components of the CCAAT-box binding factor complex (CBF/NF-Y/HAP) (Testa et al., 2005). miR169-*NFY* regulatory interactions have been indicated as operating in various plant development processes including embryogenesis and seed development (Mu et al., 2013) as well as responses to stresses (Liu and Howell, 2010; Zhao et al., 2011; Luan et al., 2014). In SE, a stress-related function of the mir169-*NF-YA10* regulatory interaction can be postulated due to the observations that stress factors affect the *TaNF-YA10* expression in wheat and that the overexpression of this gene in Arabidopsis resulted in enhanced stress tolerance (Ma et al., 2015). The stress conditions that are inevitably associated with *in vitro* cultures may also account for the differential expression of **miR319** that was indicated during SE as this molecule has been indicated as controlling the general stress-responses in Arabidopsis (Barciszewska-Pacak et al., 2015). It is possible that a mechanism of the miR319-mediated regulation of SE induction is related to auxin as miR319 was found to indirectly repress of the auxin response inhibitor, *SHY2* (*AtIAA3*) (Koyama et al., 2010). miR319 might also exert its function in SE by targeting the *TCP* (*TEOSINTE BRANCHED1/CYCLOIDEA/PROLIFERATING CELL FACTOR*) genes encoding *TFs* that are involved in the organ-specific regulation of cell growth and differentiation (Palatnik et al., 2003; Crawford et al., 2004; Nag et al., 2009). The present results showed that during the advanced stage of SE induction, which is connected with somatic embryo differentiation, miR319 appears to control two of the *TCP* genes, *TCP4* and *TCP10*.

A significantly modulated expression of numerous genes encoding auxin-responsive TFs was reported in Arabidopsis (Gliwicka et al., 2013; Wickramasuriya and Dunwell, 2015) and other plant species (Legrand et al., 2007; Sharma et al., 2008; Chakrabarty et al., 2010). Consistent with these observations, numerous miRNAs that are involved in auxin responses were identified among the candidates that are engaged in SE induction. Among them, **miR393**, which plays a key role in auxin signaling during plant development, was identified (Navarro et al., 2006; Si-Ammour et al., 2011). A recent report on SE in Arabidopsis confirmed that miR393 contributes to embryogenic transition by targeting the auxin receptors, *TIR1* and *AFB2*, and modulating tissue sensitivity to auxin treatment (Wójcik and Gaj, 2016). Another auxin signaling-related miRNA candidate, **miR160**, was indicated as controlling the development of various organs in Arabidopsis, particularly zygotic embryos, by targeting *ARF10, ARF16*, and *ARF17* (Liu et al., 2007; Liu and Chen, 2010). The down-regulation of miR160 that was observed in SE of Arabidopsis in this study was also documented in the embryogenic cultures of other plants (Zhang et al., 2012; Lin and Lai, 2013). In support of the regulatory relation between miR160 and *ARF*s during SE induction is the observation about the increased accumulation of *ARF10, ARF16*, and *ARF17* transcripts in an embryogenic culture of Arabidopsis (B. Wójcikowska and MDG., submitted for publication). In addition, the involvement of the miR160-mediated regulation of *ARF10* in the regeneration of shoots in a callus culture of Arabidopsis was also reported (Qiao et al., 2012).

Auxin/ARF-related functions can also be postulated for **miR390**, which had a significantly modulated expression in SE of Arabidopsis (present study) and of other plants (Zhang et al., 2012; Lin and Lai, 2013; Wu et al., 2015). miR390 has been documented as controlling the auxin signaling pathway by triggering the production of tasiARFs which down-regulate expression of *ARF2, ARF3*, and *ARF4* genes (Allen et al., 2005; Williams et al., 2005). The present results imply that the miR390-TAS3-ARFs regulatory interaction seems to operate during early SE induction and miR390-mediated regulation of the *ARF2* and *ARF3* genes might contribute to auxin signaling involved in embryogenic transition induced in somatic cells.

### Similarity of miRNA-mediated control in ZE and SE

The essential role of numerous miRNAs in the control of ZE was indicated in Arabidopsis and among them miR156/miR157, which are expressed in the early morphogenic stage of ZE, were identified (Nodine and Bartel, 2010; Willmann et al., 2011). In the present study, an accumulation of miR156h and miR157 was detected in the advanced stage of SE induction, which is relevant to ZE. Thus, it might be expected that, similar to ZE, these molecules control the morphogenesis of somatic embryos possibly through targeting the *SPL* (*SQUAMOSA PROMOTER BINDING PROTEIN LIKE*) genes. The present analysis of the *SPL*s vs. miR156/157 expression profiles during SE, which suggests the involvement of miR156-*SPL3/9/10/11/12/13* and miR157-*SPL2* regulatory modules in the control of somatic embryo development supports that assumption. The miRNA-mediated regulation of the *SPL* transcripts might be a common mechanism that operates during the formation of the somatic embryo as an inverse expression pattern of miR156/miR157 and *SPL* genes was also found in the SE of citrus and cotton (Wu et al., 2011; Yang et al., 2013).

The present results infer that miR164 might contribute to the SE mechanism via the regulation of the *CUC1* and *CUC2* genes encoding the CUP-SHAPED COTYLEDON transcription factors of the NAC family. Thus, similar to zygotic embryos, the miR164-*CUC1/CUC2* regulatory module appears to control establishment of the shoot apical meristem in somatic embryos (Aida et al., 1999).

The promotion of the seed maturation programme during ZE in Arabidopsis requires the miR166-mediated repression of *PHB* and *PHV* (Tang et al., 2012). Importantly for the SE induction mechanism, the *PHB* and *PHV* were reported to positively control the *LEC2*, which is the master regulator of zygotic (Stone et al., 2001) and somatic (Gaj et al., 2005; Wójcikowska et al., 2013) embryogenesis. Some evidence suggests that miR165/166 and *PHB/PHV* are involved in the LEC2-controlled pathway of SE induction since the up-regulation of the *PHB/PHV* transcript was associated with efficient SE induction and that the silencing of the *MIR166/*165 genes resulted in impaired embryogenic response (A. M. W. and M. D. G., unpublished). Similar to Arabidopsis, an inhibited expression of miR166 was attributed to the early stages of an embryogenic culture of *C. sinensis* (Wu et al., 2011), which suggests a common function of miR166 in SE induction in plants.

## Conclusions

The enrichment of the SE-related transcriptome in *MIR* transcripts that were indicated in the present study together with the extensive modulation of the *TF* genes that have been reported in embryogenic cultures of Arabidopsis and other plants confirm that a robust regulatory burst is associated with the reprogramming of plant somatic cells toward embryogenic development. The extensive modulation of *MIR* gene expression that is associated with the embryogenic transition appears to be distinctly controlled at the post-transcriptional level and as a result, the final level of mature miRNA, causative for SE induction, is adjusted.

The functions annotated to the SE-involved miRNA candidates reflect a general belief about the prevalent role of stress- and hormone-related responses in the genetic mechanism that governs SE induction. In addition, notable similarities in the miRNA-mediated regulatory pathways that operate in SE to the developmental processes in ZE are evident. The results of the study provide a valuable platform for further analysis that is aimed at the identification of the miRNA-controlled regulatory pathways that contribute to embryogenic induction in plant somatic cells. Further experiments are needed to verify the involvement of the candidate miRNAs and their postulated targets in the embryogenic transition.

## Author contributions

MG, ZS, AJ, and KS conceived and designed research. KS, DB, JD, KN, AS, and AW conducted the experiments. MG and KS analyzed the data and wrote the manuscript. All the authors read and approved the manuscript.

## Funding

This work was supported by a research grant from the National Science Centre in Poland (OPUS5 2013/09/B/NZ2/03233).

### Conflict of interest statement

The authors declare that the research was conducted in the absence of any commercial or financial relationships that could be construed as a potential conflict of interest.
