# Supplementary material for: MicroRNAs Are Intensively Regulated during Induction of Somatic Embryogenesis in Arabidopsis
Source: Front Plant Sci. 2017 Jan 23;8:18. doi: 10.3389/fpls.2017.00018 (PMC5253390; doi:10.3389/fpls.2017.00018)
Supplement: Supplementary file 6 [file Table6.DOCX]

Table S6. miRNAs that were differentially expressed during SE induction and their assumed functions.

| **miRNA** | **Target family** | **Function** | **References** |
| --- | --- | --- | --- |
| **156**  **157** | SQUAMOSA-PROMOTER BINDING PROTEIN-LIKE (SPL) | timing of vegetative-phase transition and flowering | Huijser and Schmid, 2011 |
|  |  | embryo maturation | Nodine and Bartel, 2010 |
|  |  | GA response | Yu et al., 2010 |
|  |  | AUX response | Marin et al., 2010 |
|  |  | stress response | Kasschau et al. 2003 |
| **159** | MYB TRANSCRIPTION FACTORS (MYB33/65/101) | flowering time control | Achard et al., 2004 |
|  |  | leaf and flower development | Cheng et al., 2004  Millar and Gubler, 2005  Tsuji et al., 2006 |
|  |  | seed maturation | Reyes and Chua, 2007 |
|  |  | GA response | Reyes and Chua, 2007 |
|  |  | ET response | Chen et al.,2012  Zuo et al., 2012 |
|  |  | stress response | Kasschau et al. 2003 |
| **160** | AUXIN RESPONSE FACTOR (ARF10, ARF16, ARF17) | root development | Gutierrez et al., 2012 |
|  |  | AUX response | Mallory et al., 2005  Liu et al., 2007 |
|  |  | JA response | Woldemariam et al., 2012 |
|  |  | ABA response | Nonogaki, 2008 |
|  |  | stress response | Zhou et al. 2007 |
| **164** | NAC DOMAIN TRANSCRIPTION FACTOR | shoot organ development | Nag et al., 2009  Koyama et al., 2010 |
|  |  | leaf senescence | Kim et al., 2009 |
|  |  | flower developemnt | Mallory et al., 2004 |
|  |  | embryo development | Aida et al., 1997 |
|  |  | AUX response | Xie et al., 2000  Guo et al., 2005 |
|  |  | CK response | Dello Ioio et al., 2008 |
|  |  | ET response | Kim et al., 2009 |
|  |  | stress response | Kasschau et al. 2003 |
| **166** | PHAVOLUTA (PHV) TRANSCRIPTION FACTOR  PHABULOSA (PHB) TRANSCRIPTION FACTOR  REVOLUTA (REV) TRANSCRIPTION FACTOR | embryo development | Tang et al., 2012 |
|  |  | meristem development | Prigge et al. 2005 |
|  |  | stress response | Xin et al., 2010 |
| **169** | CBF/NF-Y/HAP TRANSCRIPTION FACTOR | flowering time control | Cai et al., 2007 |
|  |  | root development | Combier et al., 2006 |
|  |  | embryo development | Kwong et al., 2003 |
|  |  | ABA response | Warpeha et al., 2007 |
|  |  | stress response | Li et al., 2008 |
| **319** | TEOSINTE BRANCHED FAMILY TRANSCRIPTION FACTOR (TCP) | leaf development | Palatnik et al., 2003 |
|  |  | AUX response | Tian, 2002  Kant et al., 2009 |
|  |  | GA response | Ori et al., 2007  Yanai et al., 2011 |
|  |  | ET response | Kim et al.., 2013 |
|  |  | CK response | Liu et al., 2009 |
|  |  | stress response | Zhou et al., 2010  Liu et al., 2008  Barciszewska-Pacak et al., 2015 |
| **390** | TRANS-ACTING SIRNA3 | root development | Marin et al., 2010 |
|  | AUXIN RESPONSE FACTOR (ARF2, ARF3, ARF4) | leaf development | Adenot et al., 2006 |
|  |  | timing of vegetative-phase change | Hunter et al., 2006 |
|  |  | AUX response | Williams et al., 2005 |
|  |  | ET response | Li et al., 2004 |
| **393** | TRANSPORT INHIBITOR RESPONSE1 (TIR1) | root development | Bian et al., 2012 |
|  | AUXIN SIGNALLING F-BOX (AFB) | AUX response | Si-Ammour et al., 2011 |
|  |  | ABA response | Sunkar and Zhu, 2004 |
|  |  | stress response | Navarro et al. 2006  Chen et al., 2012 |
| **396** | (GRF) GROWTH REGULATING FACTOR | root development | Hewezi and Baum, 2012 |
|  |  | leaf development | Rodriguez et al., 2010 |
|  |  | AUX response | Hewezi and Baum, 2012  Vanstraelen and Benkova, 2012 |
|  |  | ABA response | Hewezi and Baum, 2012 |
|  |  | CK response | Hewezi and Baum, 2012 |
|  |  | ET response | Hewezi and Baum, 2012  Mizoi et al., 2012 |
|  |  | GA response | Hewezi and Baum, 2012  Varbanova et al., 2007 |
| **398** | (CSD) COPPER SUPEROXIDE DISMUTASE | stress response | Sunkar et al., 2006 |

ABA – abscisic acid, AUX – auxin, CK – cytokinin, ET – ethylene, GA – gibberelic acid,

JA – jasmonic acid
