# Supplementary material for: MicroRNAs Are Intensively Regulated during Induction of Somatic Embryogenesis in Arabidopsis
Source: Front Plant Sci. 2017 Jan 23;8:18. doi: 10.3389/fpls.2017.00018 (PMC5253390; doi:10.3389/fpls.2017.00018)
Supplement: Supplementary file 12 [file Image1.PDF]

A.

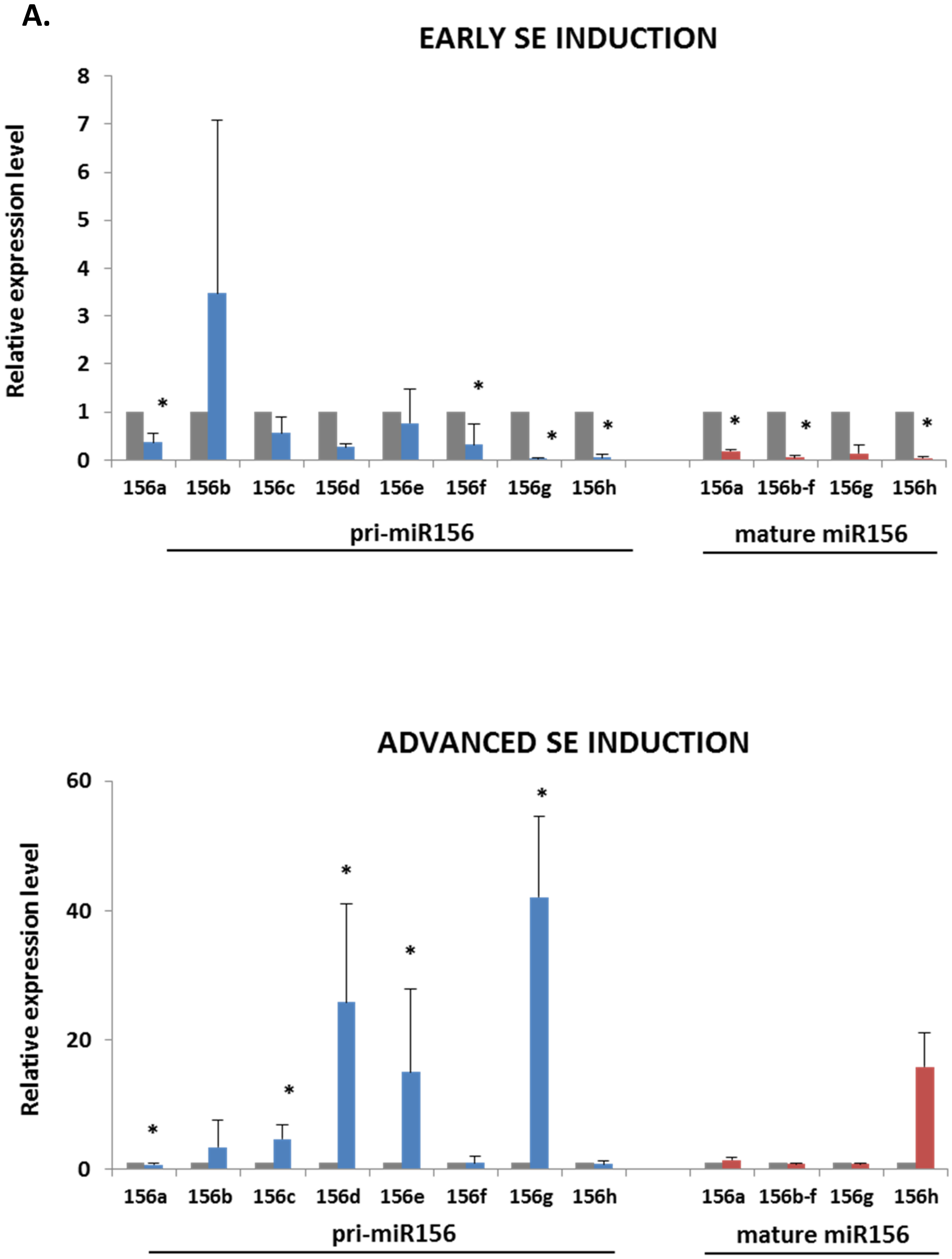
EARLY SE INDUCTION
Relative expression level
8
7
6
5
4
3
2
1
0
156a
156b
156c
156d
156e
156f
156g
156h
156a
156b-f
156g
156h
pri-miR156
mature miR156
ADVANCED SE INDUCTION
Relative expression level
60
40
20
0
156a
156b
156c
156d
156e
156f
156g
156h
156a
156b-f
156g
156h
pri-miR156
mature miR156

B.

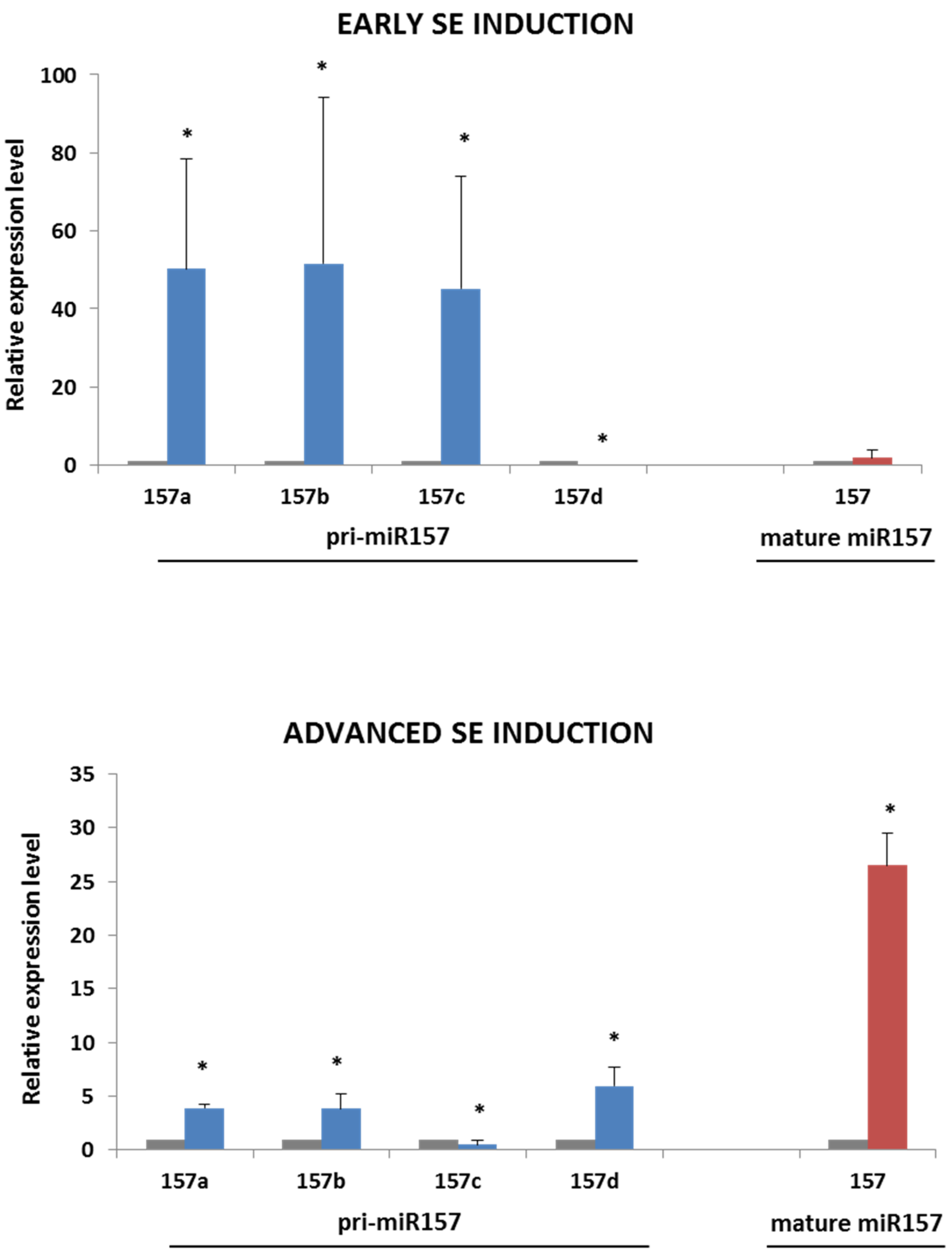
EARLY SE INDUCTION
Relative expression level
100
80
60
40
20
0
*
*
*
*
157a
157b
157c
157d
157
pri-miR157
mature miR157
ADVANCED SE INDUCTION
Relative expression level
35
30
25
20
15
10
5
0
*
*
*
*
*
157a
157b
157c
157d
157
pri-miR157
mature miR157

C.

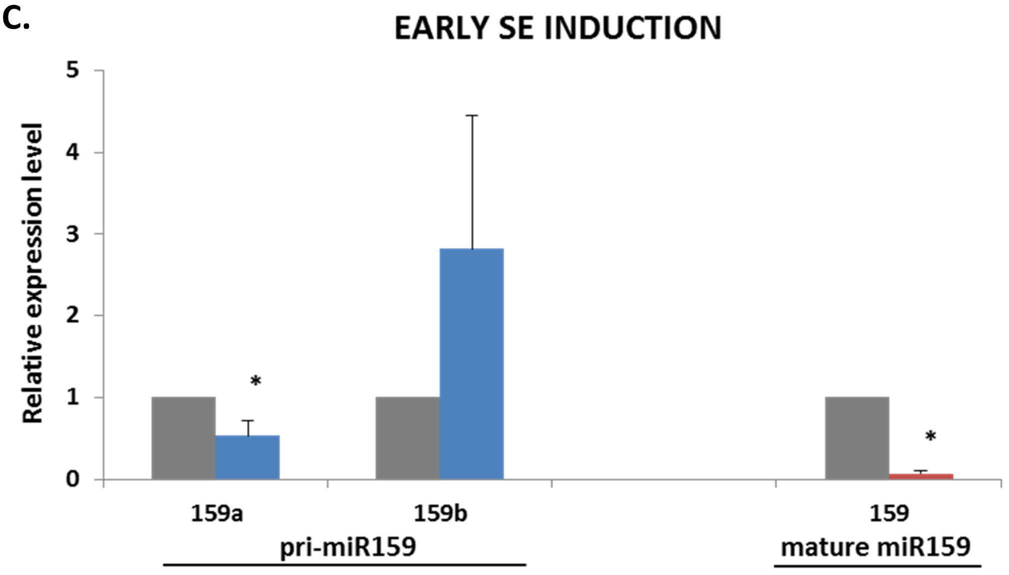

EARLY SE INDUCTION
Relative expression level
5
4
3
2
1
0
*
*
159a
159b
159
pri-miR159
mature miR159

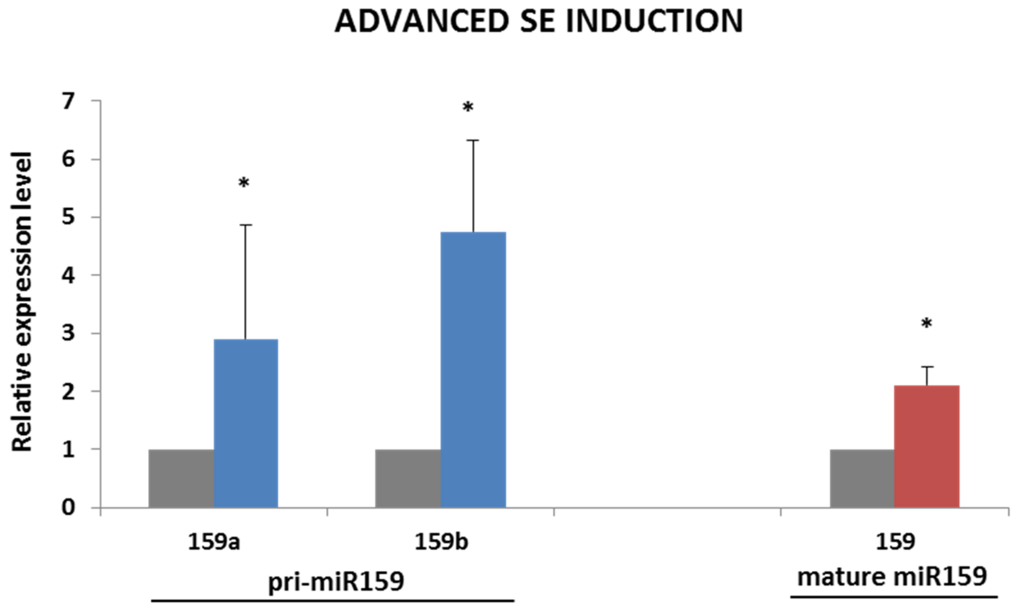

ADVANCED SE INDUCTION
Relative expression level
7
6
5
4
3
2
1
0
*
*
*
159a
159b
159
pri-miR159
mature miR159

D.

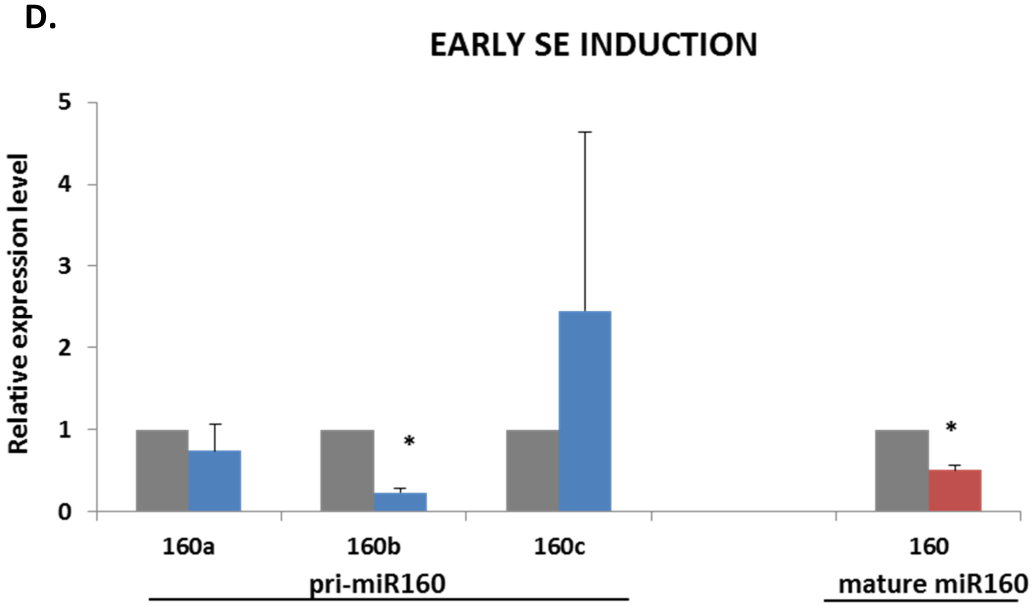

EARLY SE INDUCTION
Relative expression level
0
1
2
3
4
5
*
*
160a
160b
160c
160
pri-miR160
mature miR160

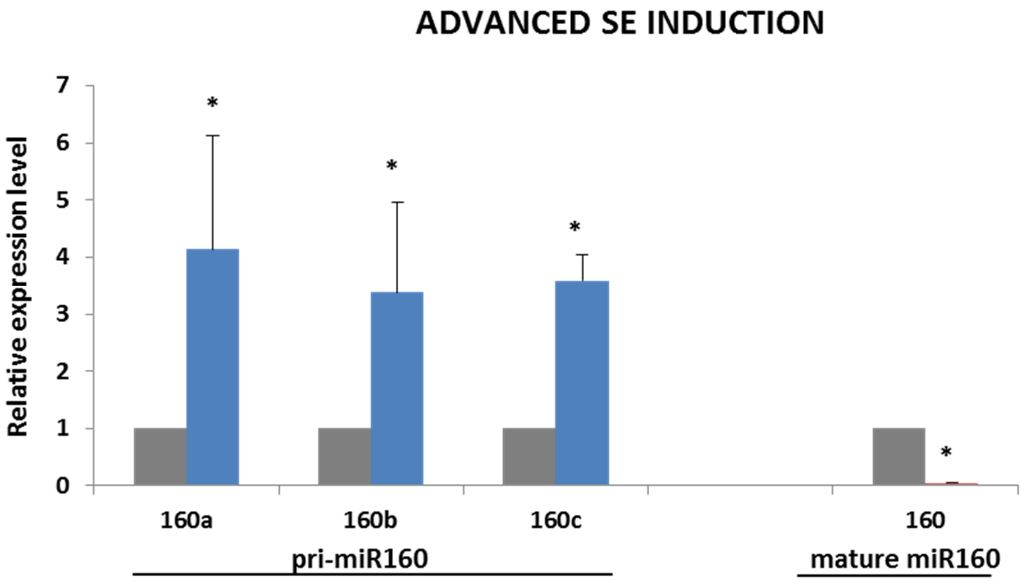

ADVANCED SE INDUCTION
Relative expression level
0
1
2
3
4
5
6
7
*
*
*
*
160a
160b
160c
160
pri-miR160
mature miR160

E.

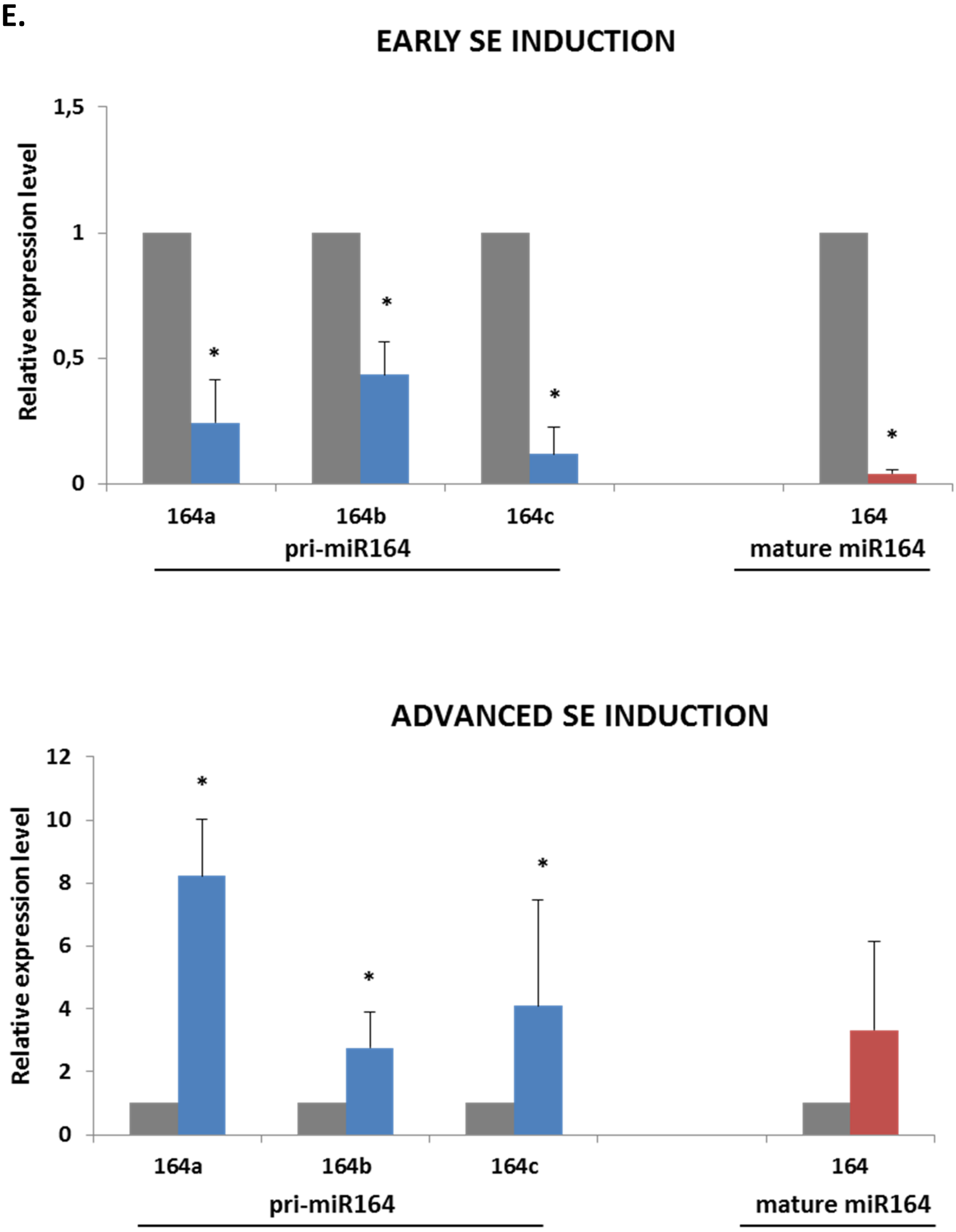
EARLY SE INDUCTION
1,5
1
0,5
0
Relative expression level
*
*
*
*
164a
164b
164c
164
pri-miR164
mature miR164
ADVANCED SE INDUCTION
12
10
8
6
4
2
0
Relative expression level
*
*
*
164a
164b
164c
164
pri-miR164
mature miR164

F.

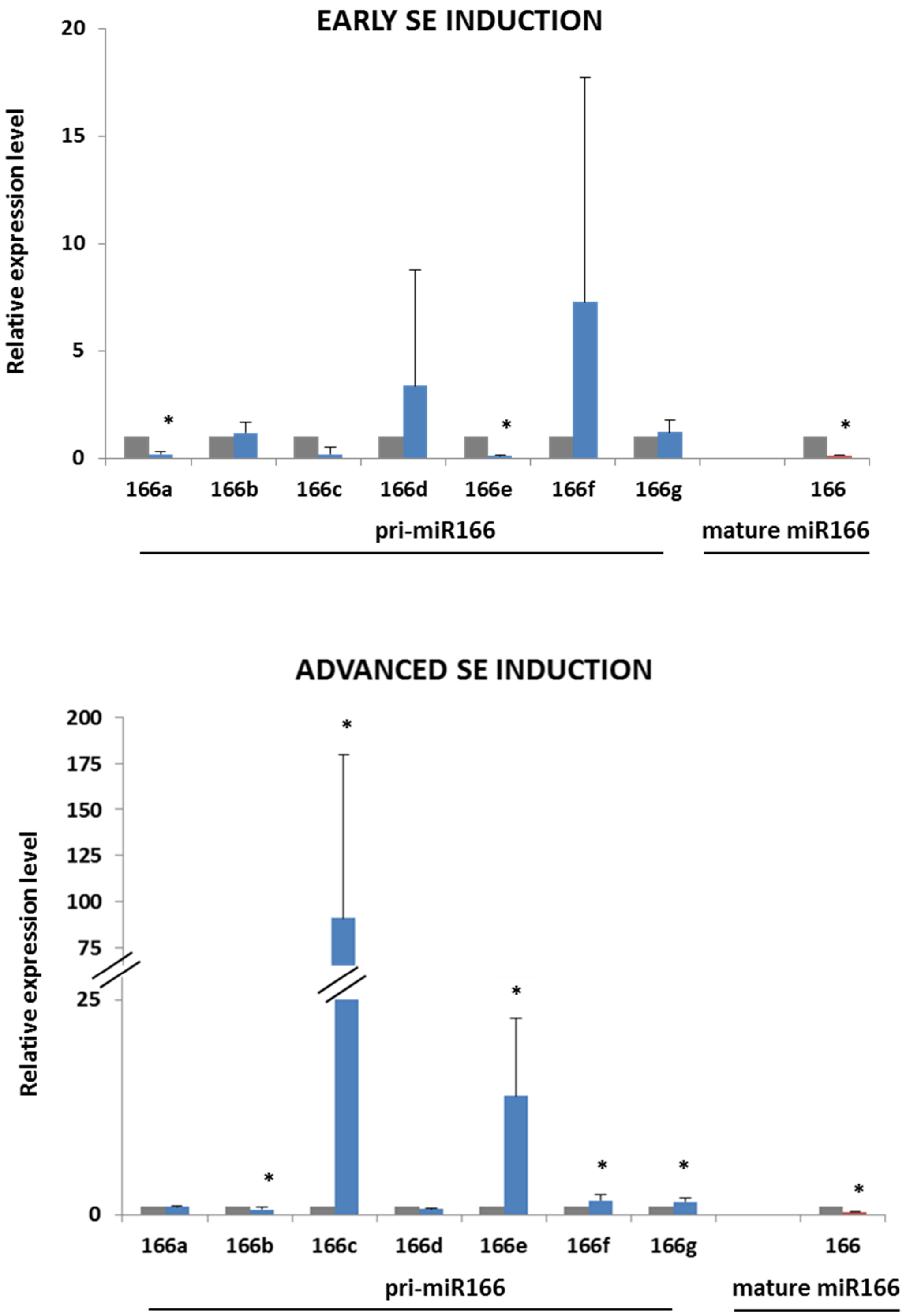
EARLY SE INDUCTION
Relative expression level
20
15
10
5
0
*
*
*
166a
166b
166c
166d
166e
166f
166g
166
pri-miR166
mature miR166
ADVANCED SE INDUCTION
Relative expression level
200
175
150
125
100
75
25
0
*
*
*
*
*
*
166a
166b
166c
166d
166e
166f
166g
166
pri-miR166
mature miR166

G.

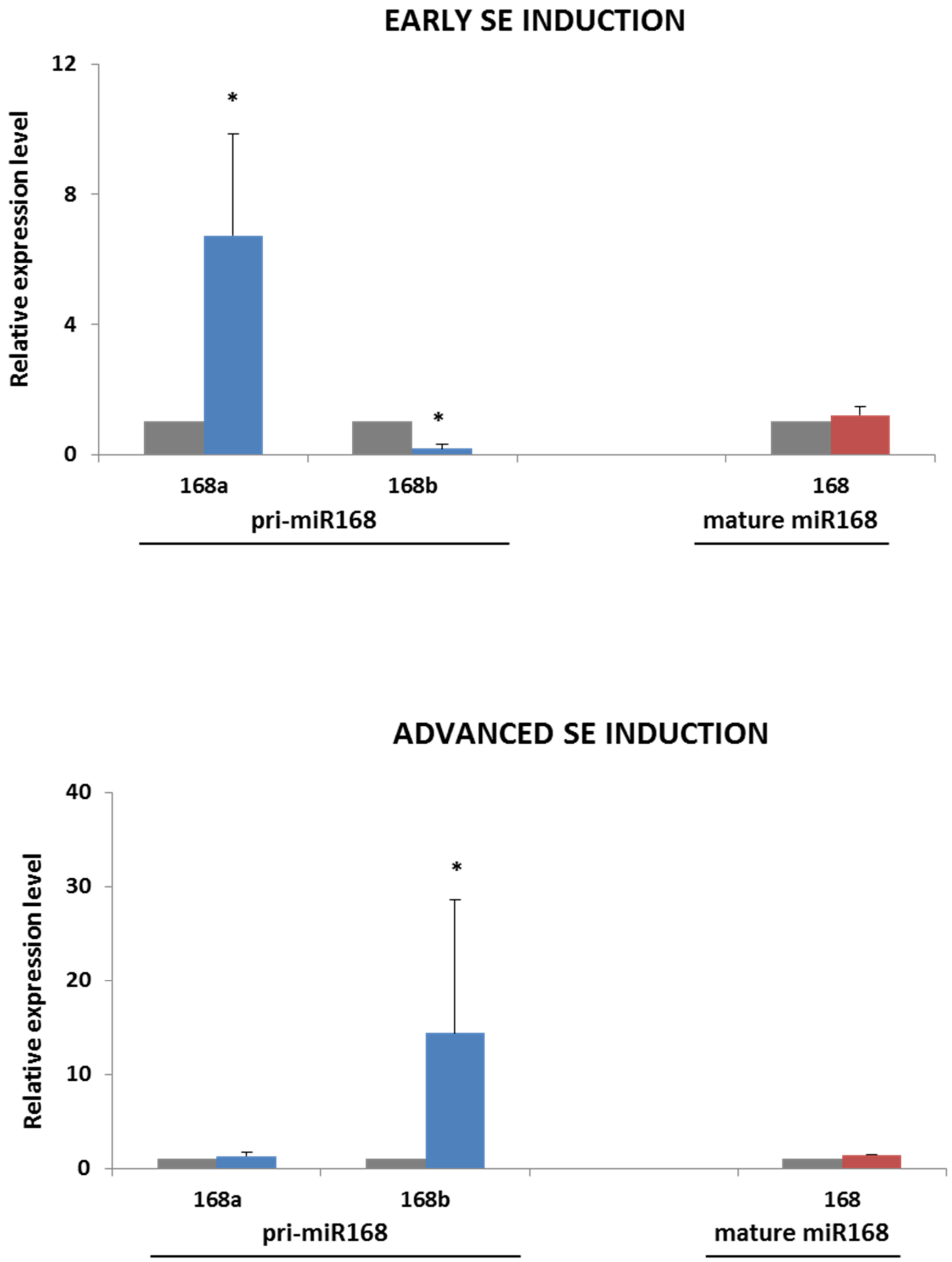
EARLY SE INDUCTION
Relative expression level
0
4
8
12
*
*
168a
168b
168
pri-miR168
mature miR168
ADVANCED SE INDUCTION
Relative expression level
0
10
20
30
40
*
168a
168b
168
pri-miR168
mature miR168

H.

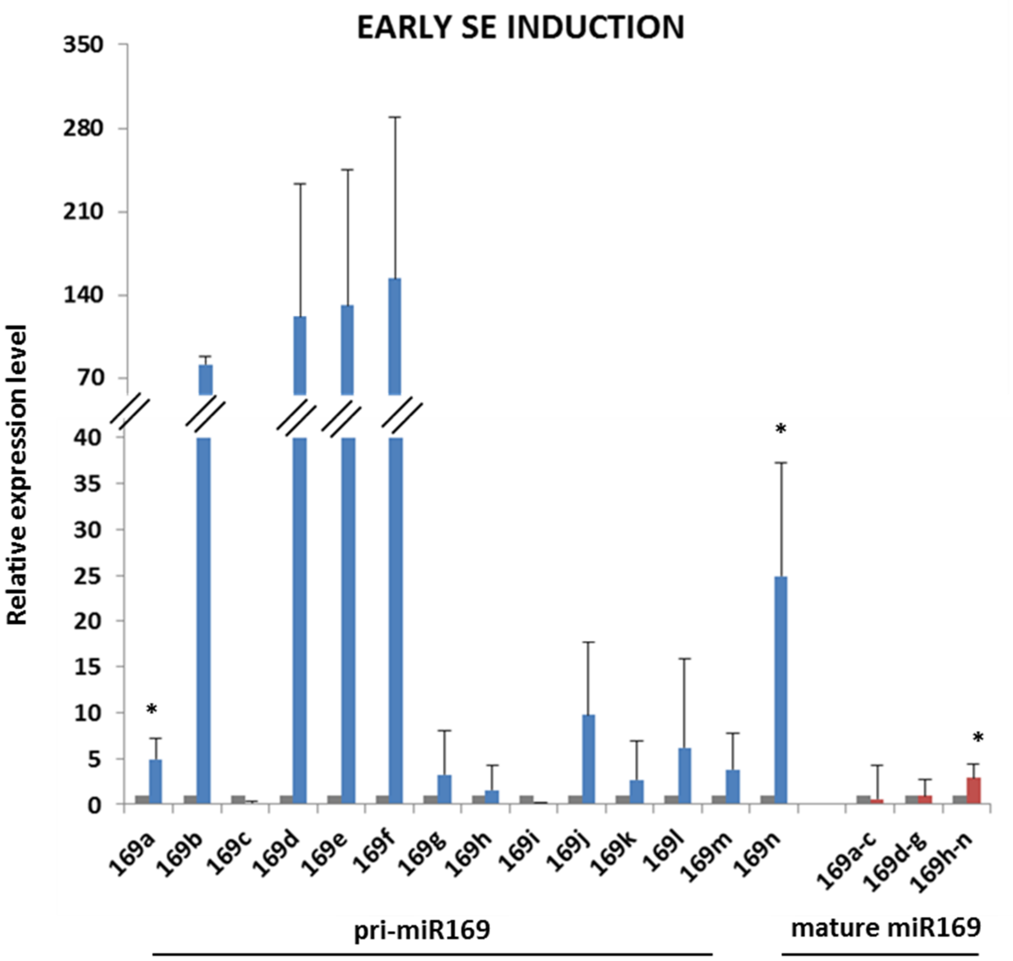

EARLY SE INDUCTION
Relative expression level
350
280
210
140
70
40
35
30
25
20
15
10
5
0
*
*
*
169a
169b
169c
169d
169e
169f
169g
169h
169i
169j
169k
169l
169m
169n
169a-c
169d-g
169h-n
pri-miR169
mature miR169

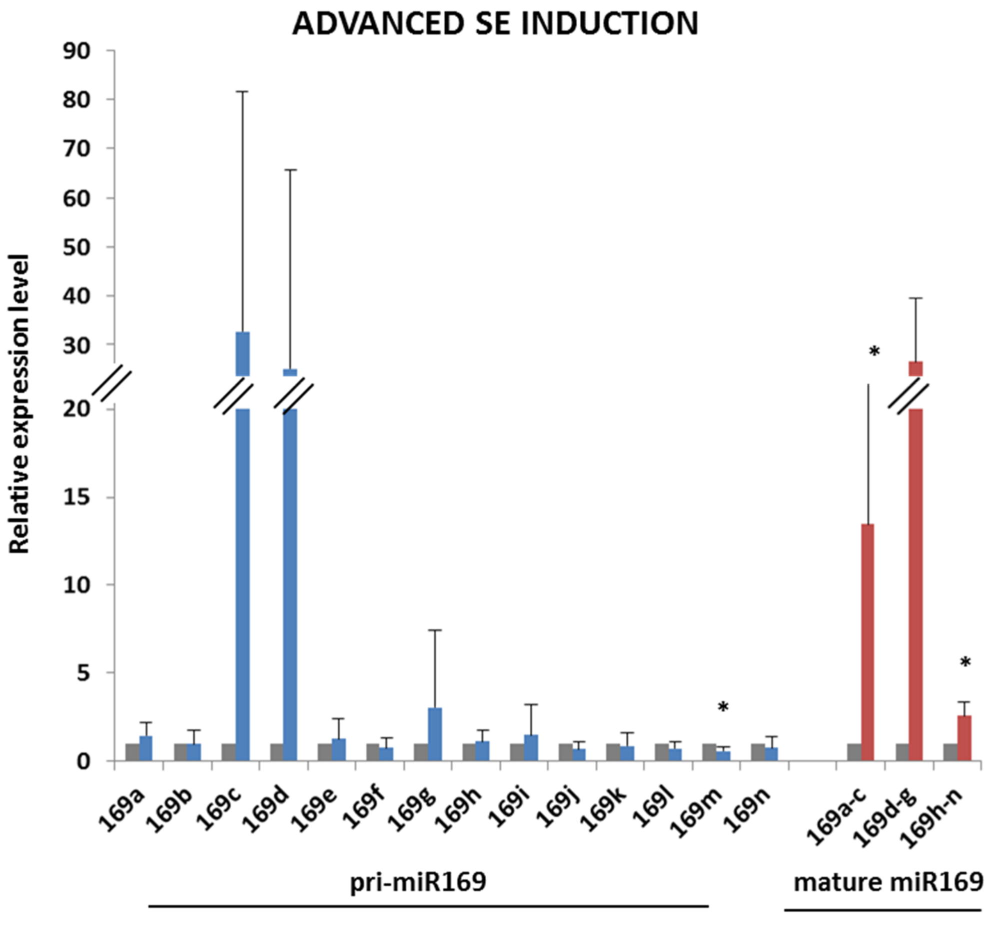

ADVANCED SE INDUCTION
Relative expression level
90
80
70
60
50
40
30
20
15
10
5
0
*
*
*
169a
169b
169c
169d
169e
169f
169g
169h
169i
169j
169k
169l
169m
169n
169a-c
169d-g
169h-n
pri-miR169
mature miR169

I.

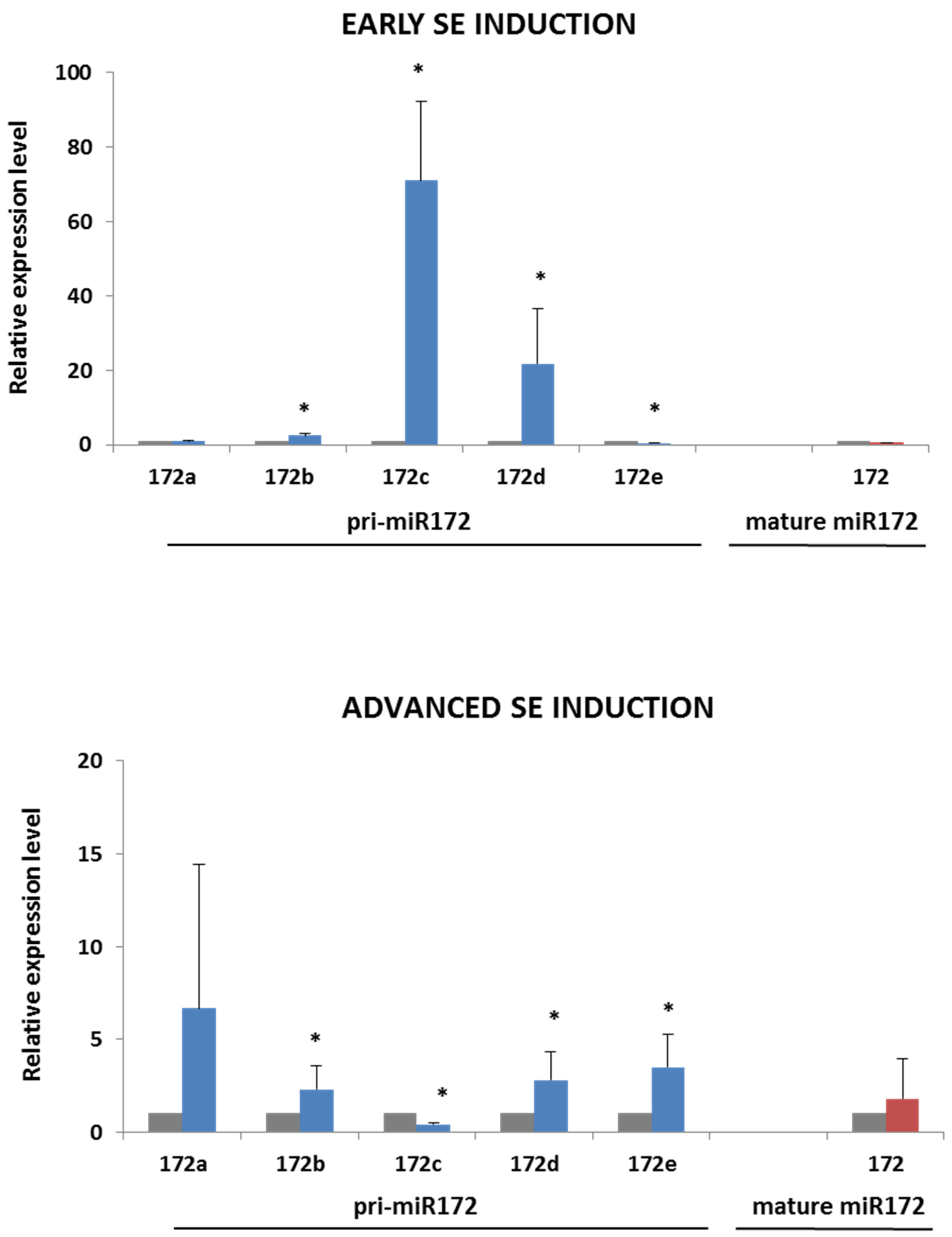
EARLY SE INDUCTION
Relative expression level
100
80
60
40
20
0
*
*
*
*
172a
172b
172c
172d
172e
172
pri-miR172
mature miR172
ADVANCED SE INDUCTION
Relative expression level
20
15
10
5
0
*
*
*
*
172a
172b
172c
172d
172e
172
pri-miR172
mature miR172

J.

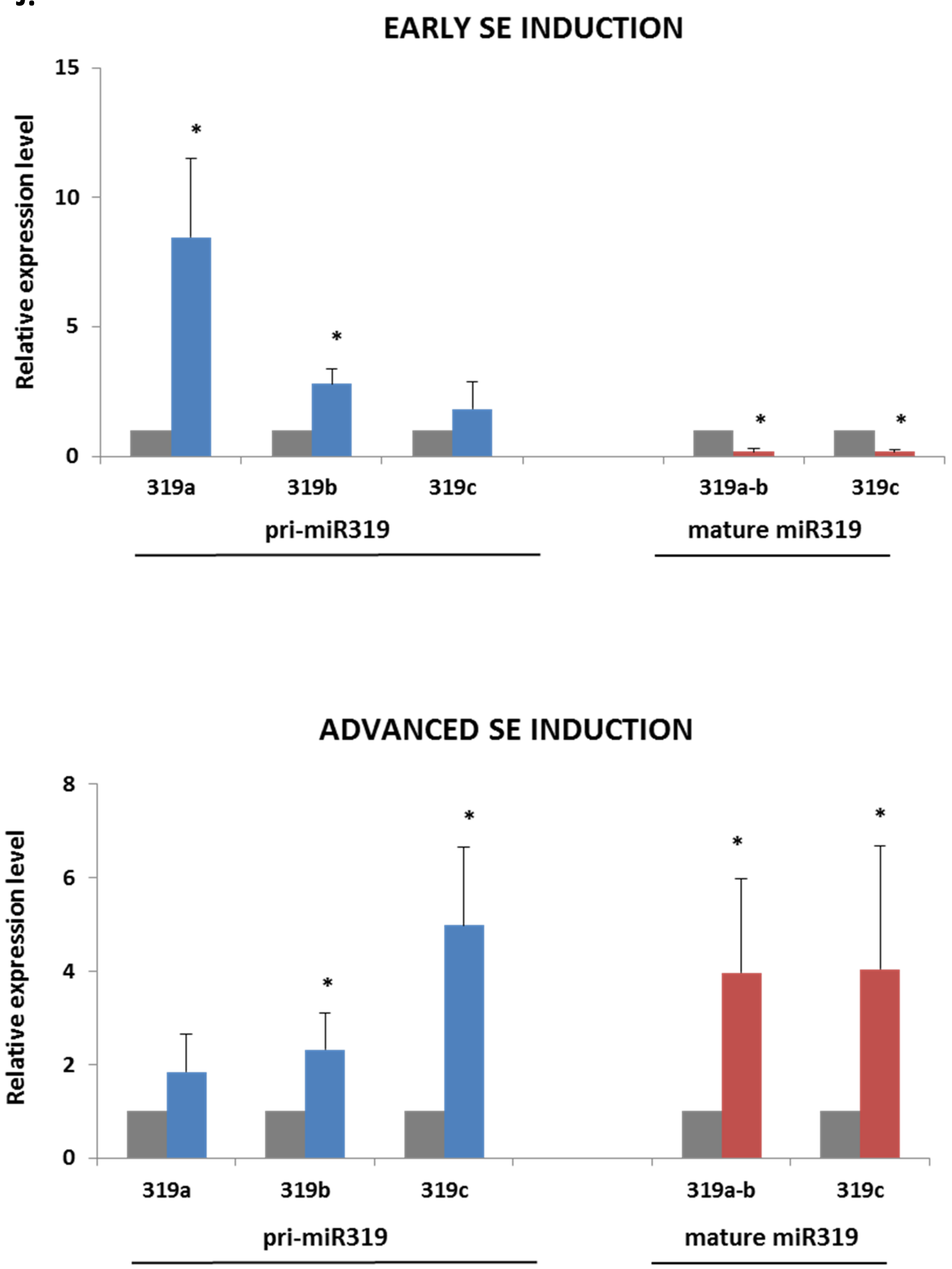

EARLY SE INDUCTION
Relative expression level
15
10
5
0
*
*
*
*
319a
319b
319c
319a-b
319c
pri-miR319
mature miR319
ADVANCED SE INDUCTION
Relative expression level
8
6
4
2
0
*
*
*
*
*
319a
319b
319c
319a-b
319c
pri-miR319
mature miR319

K.

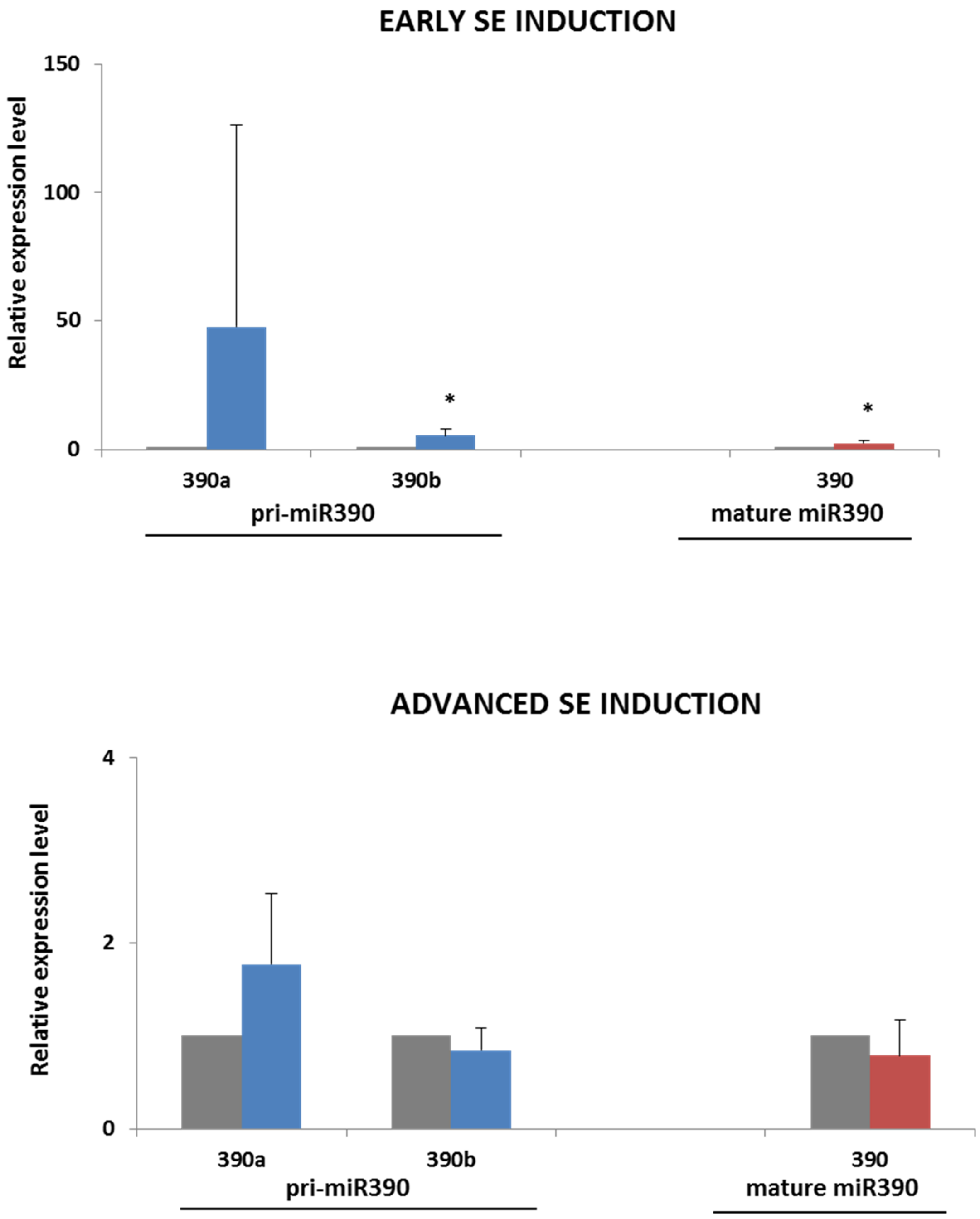
EARLY SE INDUCTION
150
100
50
0
Relative expression level
*
*
390a
390b
390
pri-miR390
mature miR390
ADVANCED SE INDUCTION
4
2
0
Relative expression level
390a
390b
390
pri-miR390
mature miR390

L.

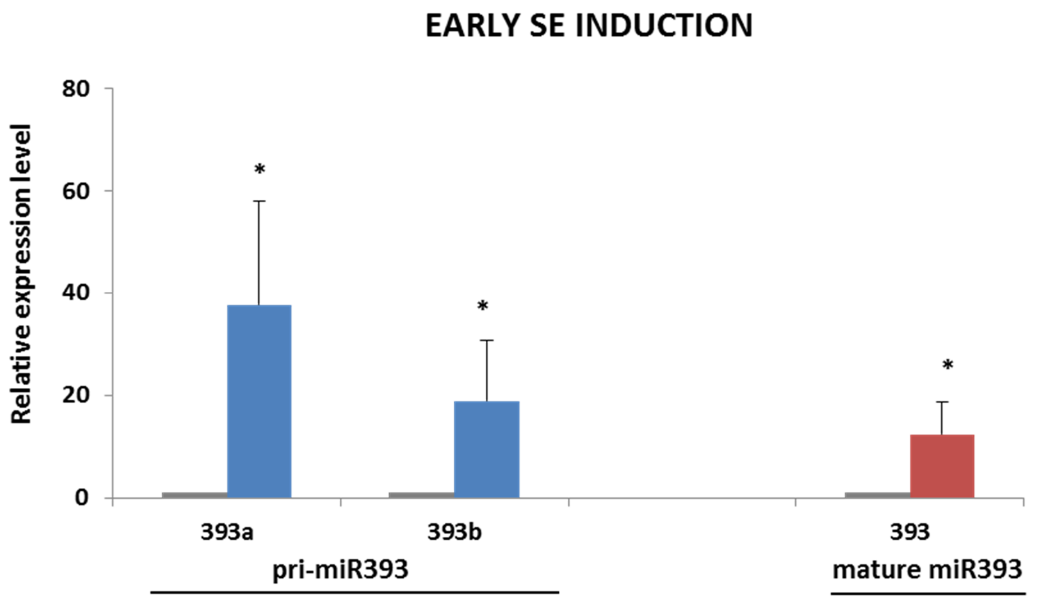
EARLY SE INDUCTION
Relative expression level
80
60
40
20
0
*
*
*
393a
393b
393
pri-miR393
mature miR393

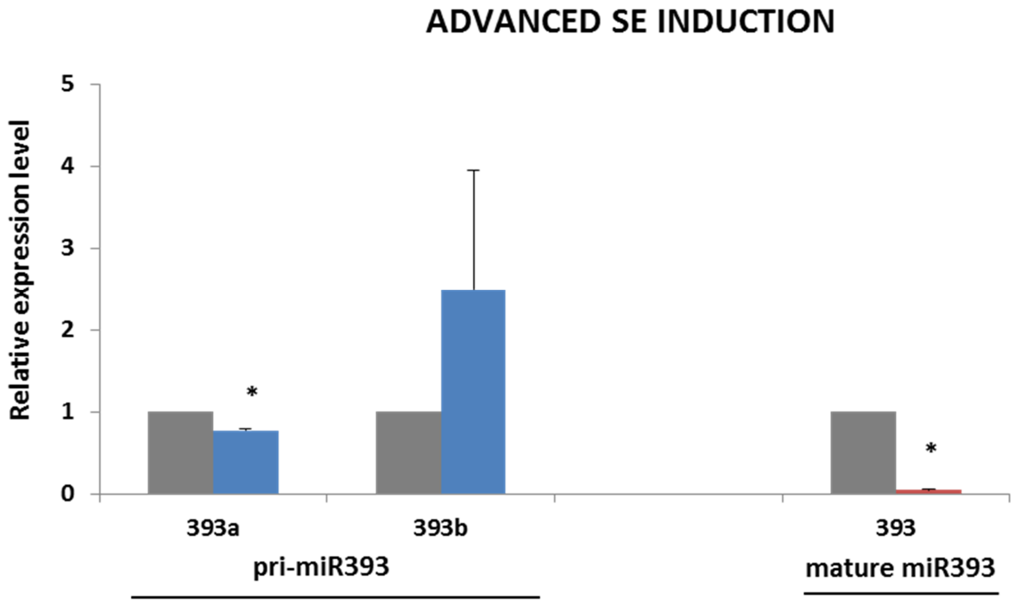
ADVANCED SE INDUCTION
Relative expression level
5
4
3
2
1
0
*
*
393a
393b
393
pri-miR393
mature miR393

M.

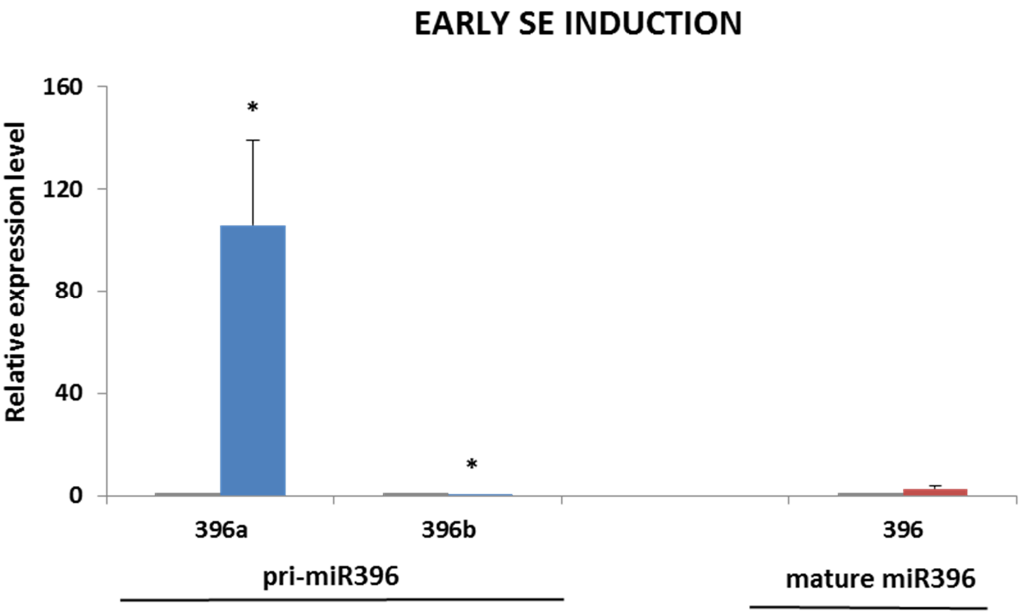

EARLY SE INDUCTION
Relative expression level
160
120
80
40
0
*
*
396a
396b
396
pri-miR396
mature miR396

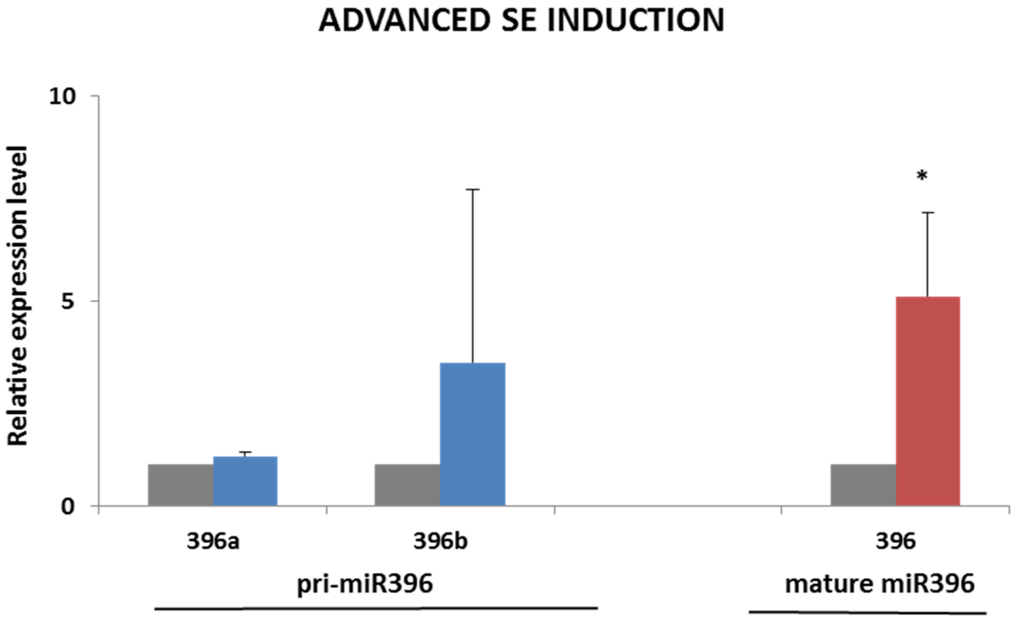

ADVANCED SE INDUCTION
Relative expression level
10
5
0
*
396a
396b
396
pri-miR396
mature miR396

N.

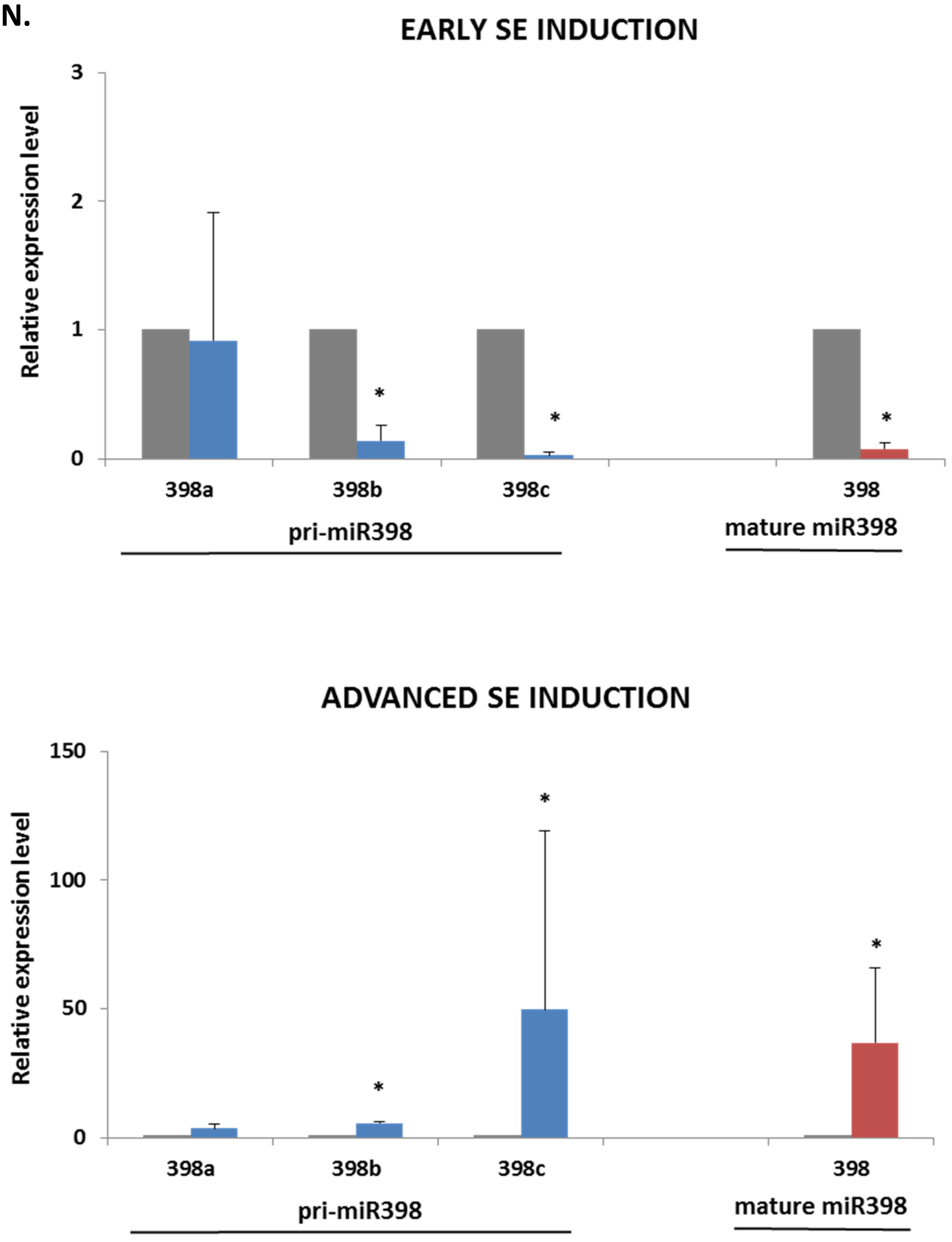

Supplementary Figure 1. Expression levels of *MIRNA* genes (blue bars) and the relevant mature miRNA (red bars) in the early (5 d – 0 d) and the advanced (10 d – 5 d) stages of SE induction. *significant difference between the compared days of SE culture ($p \leq 0.05$).
